# Transparent and Robust Artificial Intelligence-Driven Electrocardiogram Model for Left Ventricular Systolic Dysfunction

**DOI:** 10.3390/diagnostics15151837

**Published:** 2025-07-22

**Authors:** Min Sung Lee, Jong-Hwan Jang, Sora Kang, Ga In Han, Ah-Hyun Yoo, Yong-Yeon Jo, Jeong Min Son, Joon-myoung Kwon, Sooyeon Lee, Ji Sung Lee, Hak Seung Lee, Kyung-Hee Kim

**Affiliations:** 1 Digital Healthcare Institute, Sejong Medical Research Institute, Bucheon 14754, Republic of Korea; lylm@medicalai.com (M.S.L.); jangood1122@medicalai.com (J.-H.J.); kangsora39@medicalai.com (S.K.); eoao79@medicalai.com (G.I.H.); ahyoo430@medicalai.com (A.-H.Y.); yy.jo@medicalai.com (Y.-Y.J.); jmson@medicalai.com (J.M.S.); cto@medicalai.com (J.-m.K.); 2 Medical AI Co., Ltd., Seoul 06180, Republic of Korea; 3 Division of Cardiology, Department of Internal Medicine, Incheon Sejong Hospital, Cardiovascular Center, Incheon 21080, Republic of Korea; leesy@sejongh.co.kr; 4 Clinical Research Center, Asan Institute for Life Sciences, Asan Medical Center, University of Ulsan College of Medicine, Seoul 05505, Republic of Korea; totoro96a@gmail.com

**Keywords:** artificial intelligence, heart failure, electrocardiography

## Abstract

**Background/Objectives:** Heart failure (HF) is a growing global health burden, yet early detection remains challenging due to the limitations of traditional diagnostic tools such as electrocardiograms (ECGs). Recent advances in deep learning offer new opportunities to identify left ventricular systolic dysfunction (LVSD), a key indicator of HF, from ECG data. This study validates AiTiALVSD, our previously developed artificial intelligence (AI)-enabled ECG Software as a Medical Device, for its accuracy, transparency, and robustness in detecting LVSD. **Methods:** This retrospective single-center cohort study involved patients suspected of LVSD. The AiTiALVSD model, based on a deep learning algorithm, was evaluated against echocardiographic ejection fraction values. To enhance model transparency, the study employed Testing with Concept Activation Vectors (TCAV), clustering analysis, and robustness testing against ECG noise and lead reversals. **Results:** The study involved 688 participants and found AiTiALVSD to have a high diagnostic performance, with an AUROC of 0.919. There was a significant correlation between AiTiALVSD scores and left ventricular ejection fraction values, confirming the model’s predictive accuracy. TCAV analysis showed the model’s alignment with medical knowledge, establishing its clinical plausibility. Despite its robustness to ECG artifacts, there was a noted decrease in specificity in the presence of ECG noise. **Conclusions:** AiTiALVSD’s high diagnostic accuracy, transparency, and resilience to common ECG discrepancies underscore its potential for early LVSD detection in clinical settings. This study highlights the importance of transparency and robustness in AI-ECG, setting a new benchmark in cardiac care.

## 1. Introduction

Heart failure (HF) shows a global prevalence of 1–2% primarily due to an aging population [1,2]. With HF associated with poor short-term and long-term survival rates, early screening and diagnosis have become increasingly critical [3]. While HF is a clinical diagnosis, echocardiography is the most widely used imaging tool for evaluating structural abnormalities and left ventricular function, including ejection fraction. However, its resource-demanding nature has spurred the search for a more cost-effective and accessible diagnostic method, which remains an unmet need [4]. Recent advancements include deep learning-based 12-lead electrocardiogram (ECG) models for left ventricular systolic dysfunction (LVSD) screening, sparking extensive research [5,6,7,8]. Our previous work introduced a high-accuracy deep learning-based 12-lead ECG model for LVSD detection (AiTiALVSD) [9,10,11,12,13]. 

Meanwhile, the medical artificial intelligence (AI) domain is swiftly advancing, with growing demands for not just performance but also transparency and robustness [14,15]. This shift acknowledges the intricacies of diagnostics, highlighting the need for healthcare professionals to navigate AI applications in medicine with confidence [16]. While such technologies have rapidly progressed in the field of medical imaging, AI-ECG analysis still lags behind—especially in aspects of transparency [17,18,19]. Addressing this, we have developed a dedicated dataset and embarked on research focusing on reliability and clinical applicability. Our approach includes diverse, comprehensive, and quantitative evaluations of AI-ECG analysis, aiming to bridge the current research gap [4,20,21]. Through this effort, we propose systemic methods for both quantitative and qualitative assessment of AI-ECG model transparency and robustness.

## 2. Methods

### 2.1. Study Design

**Figure 1:**
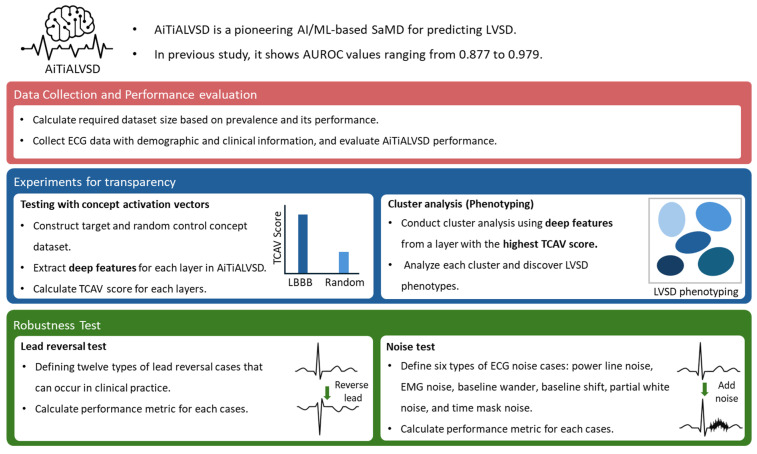
Study flow for evaluation of AiTiALVSD. LVSD, left ventricular systolic dysfunction; AI, artificial intelligence; ML, machine learning; SaMD, Software as a Medical Device; ECG, electrocardiogram; AUROC, area under the receiver operating characteristic curve; LBBB, left bundle branch block; TCAV, testing with concept activation vectors; EMG, electromyography.

This retrospective, single-center cohort study analyzed data from a pivotal clinical study on AI/ML-enabled Software as a Medical Device (SaMD) for ECG analysis, named AiTiALVSD. The original cohort was established in the Republic of Korea to support regulatory approval from the Korean Ministry of Food and Drug Safety (MFDS), and the present analysis represents a post hoc evaluation of that pivotal study. The study’s objective was to evaluate AiTiALVSD’s diagnostic performance, transparency, and robustness by juxtaposing ECG analysis outcomes with echocardiographic data, the reference standard, in patients 18 years or older suspected of LVSD at Mediplex Sejong Hospital in the Republic of Korea, spanning March 2017 to August 2021. Figure 1 shows study flow for evaluating ECG SaMD.

It outlines the methodology for validating the AiTiALVSD, a pretrained model for predicting left ventricular systolic dysfunction (LVSD). Initially, data collection protocols are established to gather a statistically significant sample and relevant demographic and clinical data. The model’s performance is then evaluated by selecting appropriate metrics and comparison targets. Transparency is addressed through experiments involving concept activation vectors (TCAV) and cluster analysis, which are used to understand model decisions and discover LVSD phenotypes. Finally, robustness tests are conducted, including a reverse test to assess performance under various lead reversal cases, and a noise test to evaluate the model’s resilience to different types of ECG noise. This structured process aims to ensure the model’s performance, transparency, and robustness for application in real-world.

The inclusion and exclusion criteria described below were originally defined as part of the pivotal MFDS study. Inclusion criteria required a 12-lead ECG taken within 24 h prior to echocardiography, a 10 s measurement duration, and a sampling rate of 500 Hz using a PAGEWRITER TC30 or TC70 (Philips, Cambridge, MA, USA). If multiple ECGs were available, the one closest in time to the echocardiography was chosen. Exclusion criteria included missing ECG data, gaps in recording, or lack of echocardiography to confirm left ventricular ejection fraction (LVEF). This study received approval for the investigational device exemption (IDE) trial from the institutional review boards of Mediplex Sejong Hospital (IRB File No. ISH-2022-09-005-003), with informed consent waived due to its retrospective study design. To minimize potential bias, all data were independently collected and managed by third-party clinical sites, ensuring no direct involvement from the software development team. Additionally, the analysis was conducted in a blinded manner, with evaluators unaware of patient identifiers or other contextual information that could influence their assessments.

### 2.2. Variables and Reference Standards

Patient demographic and clinical profiles, including age, sex, baseline characteristics, and lab results, were retrieved from electronic records, with LVEF assessed via the biplane Simpson method. LVSD was identified with an EF ≤ 40%, contrasting a non-LVSD definition of EF > 40%. Reference standard data was anonymized to maintain confidentiality. An independent evaluator, blind to LVSD status, input the 12-lead ECG data into AiTiALVSD, documenting the algorithm-determined LVSD scores and risk categories.

### 2.3. AI-ECG Model Description

AiTiALVSD, an AI/ML-based SaMD, exclusively analyzes 12-lead ECG raw data to aid in diagnosing LVSD, determining if the LVEF is 40% or lower. Upon processing ECG data, it outputs a 0–100 score reflecting LVSD likelihood. Scores ≥ 9.7, set for 90% sensitivity from prior research, indicate high LVSD probability, while scores below this mark suggest low risk [9].

Developed around a one-dimensional residual neural network (ResNet) using the PyTorch framework (version 2.0.1) and Python, AiTiALVSD features a composition of a stem block, four feature blocks, and a fully connected layer for LVSD pattern recognition [22,23]. Each feature block, a series of three residual blocks, sequentially processes and extracts ECG characteristics for analysis. To standardize input dimensions and improve computational efficiency, all ECG data were resampled to 250 Hz. ECG data originally recorded at 500 Hz were downsampled accordingly. Each 10 s ECG was thus represented as a 12 × 2500 array and used as the input for binary LVSD classification. All other experiments were conducted using the same preprocessing procedures. Further model development details, data preprocessing, and data distribution specifics are available in Supplementary Texts S1 and S2.

### 2.4. Experiments for Transparency

In the clinical domain, significance of explainable AI has escalated, emphasizing two critical aspects: clinical plausibility and exploitation [24]. Clinical plausibility confirms that AI-generated outcomes are reliable and relevant in clinical environments. In contrast, exploitation refers to the application of these findings to corroborate existing medical insights. Whereas previous research predominantly concentrated on local interpretative methods, which clarify the rationale behind AI predictions for singular data points by spotlighting key features, this approach inadequately addresses the explanation of specific characteristics, Conversely, global interpretative strategies strive to demystify the overarching rationale of AI models across diverse inputs, providing an integral perspective of their predictive logic [4,21].

1. Testing with Concept Activation Vectors

The implementation of testing with concept activation vectors (TCAV) allows for the assessment of AiTiALVSD’s consistency with medically recognizable concepts [25]. TCAV scores, derived from this evaluation, quantify the influence of specific concepts on AiTiALVSD’s feature blocks during prediction. These scores, ranging from 0 to 1, signify the degree of positive impact a concept has on predicting LVSD, with scores near 1 indicating significant influence. Utilizing the Physionet Challenge 2021 dataset, we constructed nine “LVSD concept” datasets from 26 different ECG interpretation labels: atrial fibrillation and atrial flutter (AF), left bundle branch block (LBBB), right bundle branch block (RBBB), left axis deviation (LAD), right axis deviation (RAD), prolonged QT, conduction disorder, abnormal Q wave, and abnormal T wave [26]. The AF concept included both atrial fibrillation and atrial flutter. The abnormal T wave concept included both abnormal T wave and T wave inversion labels. These “LVSD concepts” represent ECG indicators recognized as heart failure with reduced ejection fraction risk factors, supported by recent studies [27,28]. TCAV scores are presented with a 95% confidence interval (CI) for precision, detailed further in Supplementary Text S3. For analysis, we utilized the captum python package (version 0.6.0), with TCAV research code for ECG available at: https://github.com/medicalai-research/TCAV_ECG (accessed on 1 July 2024) [29].

2. Cluster analysis

Our goal was to phenotype LVSD by clustering AiTiALVSD’s hidden features, especially those within the block demonstrating the highest TCAV score. We posited that these prominent features would most accurately reflect ECG characteristics in alignment with established medical knowledge. Through a comparative analysis of demographic, electrocardiographic, and echocardiographic features across clusters, we verified AiTiALVSD’s capability to discern key LVSD traits. Employing positively predicted ECGs from a previous development dataset, we trained a K-means clustering algorithm, subsequently applying the model to our dataset [30]. The optimal cluster count was ascertained using the elbow algorithm. Detailed methodologies are elaborated in Supplementary Text S3.

### 2.5. Tests for Robustness

Acknowledging the quality variance between data from controlled experiments and real-world clinical scenarios, we initiated robustness assessments to preemptively identify and mitigate potential model performance discrepancies due to data quality changes.

1. Lead reversal test

To assess our model’s resilience against electrode misplacement, we generated potential 12-lead ECG morphologies for each patient, resulting in an evaluation of the model across 12 distinct types of lead reversal scenarios from a pool of 681 ECGs [31]. Despite the 24 conceivable lead reversal permutations, only 12 unique morphologies were identified due to identical presentations in certain pairs, e.g., [Nonreversal] or [LL/RL reversal], highlighting the model’s diagnostic accuracy in identifying LVSD under varied electrode configurations.

2. Noise robustness test

Our noise resilience test incorporated six types of artificial noise: power line, electromyography (EMG), baseline wander and shift, partial white noise, and time mask noise, reflecting common distortions in real ECG data. By applying these noise profiles to our dataset, we observed AiTiALVSD’s performance shifts, identifying critical noise factors impacting accuracy. This comprehensive approach enables the determination of AiTiALVSD’s noise robustness, as detailed in Supplementary Figure S1 and validated across the modified datasets.

### 2.6. Statistical Analysis 

Data were analyzed using appropriate statistical tests based on the variables type and distribution. Student’s *t* test or Mann–Whitney U test were used for comparing continuous variables, depending on normality. Categorical variables were compared using the Chi-square test or Fisher’s exact test, as appropriate. Correlation analysis was performed using Pearson correlation methods. Model performance was assessed by calculating the area under the receiver operating characteristic curve (AUROC). We determined the AiTiALVSD threshold point that gave a sensitivity ≥90% (AiTiALVSD score 9.7) during model development. We report this threshold’s sensitivity, specificity, negative predictive value (NPV), and positive predictive value (PPV), along with 95% CI. We confirmed the AUROC with both continuous predictors and binary predictors. AUPRC was also estimated to the model performance, using only a continuous output variable. For the lead reversal and noise robustness analyses, AUROC and AUPRC were estimated based on the continuous output variable. Subgroup analyses were performed by demographics, medical history, several ECG patterns, and NT-proBNP. To adhere to the conditions set by Mediplex Sejong Hospital, patients with NT-pro BNP test results equal to or greater than 158 pg/mL (ARCHITECT i2000 SR, Abbott, Abbott Park, IL, USA ) were classified as abnormal, while those with results below 158 pg/mL were considered normal.

In this study we utilized AiTiALVSD version 1.00.00 (Medical AI Co., Ltd., Seoul, Republic of Korea), aiming to obtain approval from the Korea Food and Drug Administration (KFDA) for a SaMD. The study was designed to meet the pivotal standards required for SaMD certification, with the performance of AiTiALVSD anticipated at an AUROC of 0.85. The criterion for clinical validity evaluation was set at an AUROC of 0.75, with the significance level (α) determined at 0.05 and the statistical power (1 − β) targeted at 95%, ensuring compliance with the stringent regulatory requirements for medical device approval in South Korea. Considering a dropout rate of 5%, the total sample size was 688 participants (103 LVSD and 585 non-LVSD). This composition reflects the prevalence of LVSD typically seen in real-world population screening settings [5,6,7,8]. We elaborated thorough backgrounds for the sample size calculation in the Supplementary Text S4. Sample size calculation was performed using PASS 15 (NCSS, LLC., Kaysville, UT, USA) software. All analyses were conducted using R (version 4.2.3) and Python (version 3.8).

## 3. Results

### 3.1. Study Population

**Figure 2:**
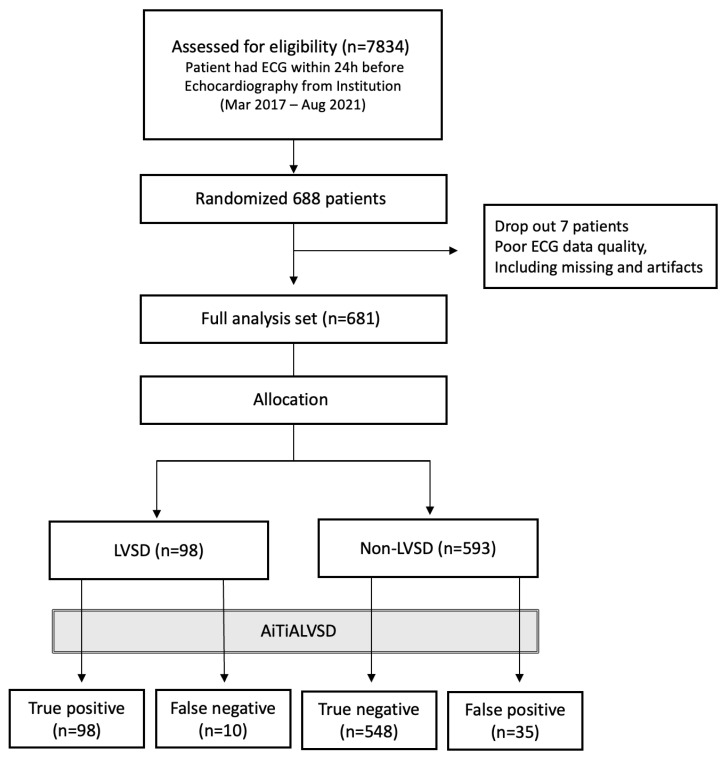
Data flow. LVSD, left ventricular systolic dysfunction; ECG, electrocardiogram.

Between March 2017 and August 2021, a total of 7834 patients who underwent both echocardiography and ECG were screened. Among them, 688 patients were randomly selected for inclusion in the pivotal clinical study’s analysis set. A total of 7 patients were excluded due to ECG data quality issues, resulting in a final study population of 681 participants. The cohort’s mean age was 60.7 ± 14.8 years, with males representing 51%. Detailed demographics and clinical profiles are in Table 1. Analysis highlighted older age and higher rates of diabetes, coronary artery disease, and chronic kidney disease in the LVSD group compared to the non-LVSD group. Data collection flow is illustrated in Figure 2.

During the study period, among 7834 ECG–echocardiography pairs that met the inclusion and exclusion criteria, 688 were selected for the eligibility. The noise assessment module filtered out 7 ECG–echocardiography pairs, and the final data of 681 pairs (98 LVSD, 593 non-LVSD) were selected as the full analysis set.

### 3.2. AI-ECG Model Performance

AiTiALVSD demonstrated a diagnostic performance for LVSD with an AUROC of 0.919 (95% CI 0.887–0.951), with its score distribution detailed in Supplementary Figure S2. The LVSD group’s scores predominantly clustered below 5.0, peaking at 53.5. Notably, AiTiALVSD scores inversely correlated with LVEF values, evidenced by a Pearson R of −0.781 (*p* < 0.001), as shown in Supplementary Figure S3.

Performance metrics outlined in Table 2 reveal AUROC, sensitivity, specificity, PPV, and NPV scores of 0.971, 0.898, 0.940, 0.715, and 0.982, respectively. Performance differentiation for EF thresholds of 35% and 50% showed AUROCs of 0.906 (95% CI 0.869–0.943) and 0.891 (95% CI 0.857–0.925), respectively, detailed in Supplementary Table S1.

Subgroup analyses, accounting for variations in clinical characteristics and ECG patterns, demonstrated consistent performance, with AUROCs generally above 0.7. Although some subgroups such as atrial fibrillation and chronic kidney disease had wider confidence intervals due to smaller sample sizes, the model retained acceptable discrimination in all categories (Supplementary Table S2). Supplementary Tables S3 and S4 contrast the performances of NT-proBNP and AiTiALVSD. The AUROC values were 0.72 (95% CI 0.635–0.804) and 0.905 (95% CI 0.842–0.968), respectively, while the sensitivity was 0.889 (95% CI 0.770–1.0) for NT-proBNP and 0.926 (95% CI 0.827–1.0) for AiTiALVSD among a subset of 96 patients.

### 3.3. TCAV Interpretation

**Figure 3:**
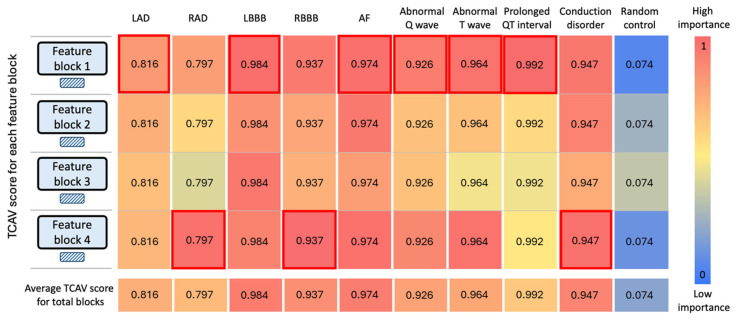
Testing with concept activation vectors of AiTiALVSD. The red frame indicates the block with the highest TCAV score for each concept. LVSD, left ventricular systolic dysfunction; ECG, electrocardiogram; LAD, left axis deviation; RAD, right axis deviation; LBBB, left bundle branch block; RBBB, right bundle branch block; AF, atrial fibrillation.

Figure 3 and Supplementary Table S8 illustrate TCAV’s analysis across AiTiALVSD’s four feature blocks, demonstrating significant engagement with nine LVSD-associated concepts. These concepts, notably LBBB, RBBB, AF, and conduction disorder, achieved average TCAV scores exceeding 0.8, highlighting their integral role in LVSD prediction. This pattern suggests AiTiALVSD not only accurately identifies these cardiac conditions but also prioritizes their importance in diagnosing LVSD. Each TCAV score is presented alongside a 95% confidence interval derived from repeated directional derivative testing to assess statistical significance. For instance, the TCAV score for LBBB was 0.984 with a 95% CI of 0.978–0.990, indicating a strong and statistically significant influence on model predictions.

Particularly, the first feature block showed superior TCAV scores for six concepts (LAD: 0.816, LBBB: 0.984, AF: 0.974, abnormal Q wave: 0.926, abnormal T wave: 0.964, and prolonged QT: 0.922), suggesting a heightened extraction of relevant features for LVSD prediction compared to other blocks. This emphasizes the first block’s extraction of clinically significant features, laying the groundwork for subsequent cluster analysis utilizing these characteristics.

It shows TCAV results extracted from the four feature blocks that make up AiTiALVSD. Based on existing medical knowledge, we determined the nine concepts of ECG features corresponding to LVSD high risk and analyzed their impact on LVSD detection by comparing them with a random feature concept that excludes the features; in the first and last blocks, the random features had little importance, but the nine concept features had high relative importance. 

### 3.4. Clustering of AiTiALVSD-Positive LVSD Cases for Phenotyping 

**Figure 4:**
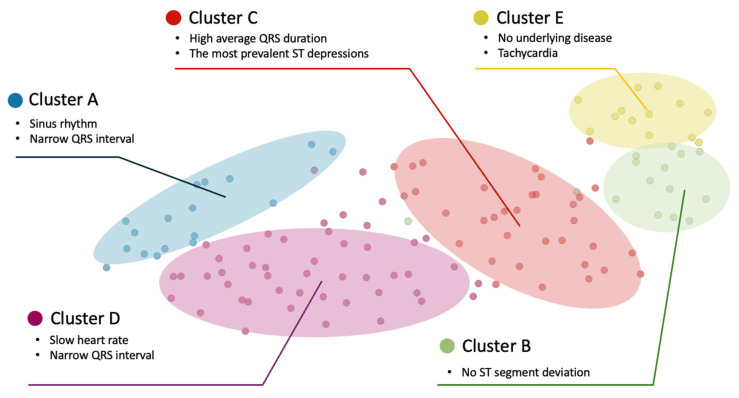
Visualization of clustering analysis using t-distributed stochastic neighbor embedding (T-SNE).

Leveraging TCAV insights, we hypothesized that the first feature block’s hidden features would closely align with established electrocardiographic knowledge. This premise guided our cluster analysis of LVSD-positive electrocardiograms identified by AiTiALVSD, resulting in five distinct clusters within our development dataset, facilitating a detailed demographic, electrocardiographic, and echocardiographic feature comparison among clusters (Figure 4 and Supplementary Text S1). This analysis extended to lead-specific Q and ST-T abnormalities across all 12 leads (Supplementary Table S5).

This figure presents a two-dimensional scatter plot illustrating five distinct clusters, each representing a different ECG characteristic within a patient cohort. The clusters are differentiated by colors and labeled from A to E, with each cluster denoting unique ECG features.

In our 681-participant clinical trial, AiTiALVSD predicted 123 individual LVSD phenotypes, distributed into five clusters: Cluster A (16 individuals) exhibited a predominant sinus rhythm, narrow QRS intervals, notable abnormal Q waves in anterior (V2-4) and septal leads, and a significant T wave inversion in anterior and lateral leads. This cluster, with an 81.25% true positive rate, had a history of hypertension, coronary artery disease, and chronic kidney disease, with average AiTiALVSD scores and LVEF of 42.9 and 36.7%, respectively. Cluster B (13 ECGs), with the least CAD and CKD prevalence, displayed no ST segment deviations, a common QT prolongation, and ST elevation, with the lowest average AiTiALVSD score of 33.2 and an LVEF of 41.4%. Cluster C (32 ECGs), noted for its high QRS duration and T inversion rate in inferior leads, showed the most ST depressions and the highest LVH interpretation by ECG devices, alongside the longest E/E’ and E/A ratios. This cluster had the highest AiTiALVSD score of 55.87 with an LVEF of 35.97%. Cluster D (49 ECGs), characterized by high CAD prevalence, had the lowest mean heart rate, shortest QTc interval, a presence of pacemakers, and similar Q wave abnormalities to Cluster A, with average LVEF and AiTiALVSD scores of 39.3% and 40.0, respectively. Cluster E (13 individuals) showed the lowest prevalence of past medical conditions, highest heart rate, shortest QRS duration, highest RAD occurrence, and significant AF presence, with average LVEF and AiTiALVSD scores of 38.1% and 37.7, respectively. Supplementary Figures S5–S9 display representative true positive ECGs for each cluster.

### 3.5. Lead Reversal Analysis

**Figure 5:**
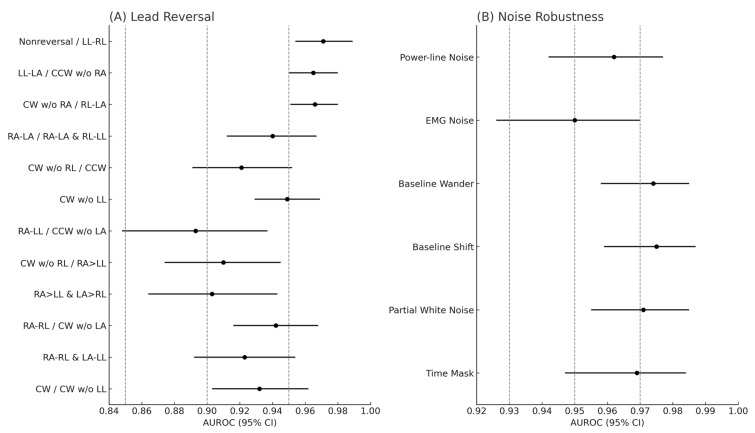
Forest plots of AiTiALVSD performance under lead reversal and ECG noise conditions. (**A**) Diagnostic performance of AiTiALVSD across 12 types of ECG lead reversal scenarios. Each dot represents the mean AUROC, with horizontal bars indicating the 95% confidence intervals. Vertical dashed lines at AUROC values of 0.85, 0.90, and 0.95 serve as visual guides. (**B**) Diagnostic performance of AiTiALVSD under six types of ECG noise conditions, presented similarly. The model demonstrated consistently high AUROC values in most scenarios, confirming its robustness across both technical and environmental variability. AUROC, area under the receiver operating characteristic curve.

Within the 681-case full analysis set, 12 unique ECG lead reversal configurations were examined, with performance outcomes detailed in Figure 5A and Supplementary Table S6. AiTiALVSD consistently demonstrated high diagnostic accuracy, achieving an AUROC of over 0.9 for all but the ‘RA/LL reversal and counter-clockwise reversal without LA’ scenario. Furthermore, the model maintained a sensitivity rate above 80% across these conditions, highlighting its reliable detection capabilities even amidst lead reversals.

### 3.6. Noise Robustness Analysis

Figure 5B and Supplementary Table S7 present AiTiALVSD’s performance under various noise influences, such as power line interference, baseline wander and shift, partial white noise, and time mask, demonstrating resilience by showing statistical consistency with clean ECG data, as indicated by overlapping 95% CI across performance metrics. However, exposure to EMG noise resulted in a notable drop in specificity (from 0.940 to 0.847) and positive predictive value (from 0.715 to 0.508), indicating reduced ability to accurately identify negative cases under this noise type.

## 4. Discussion

This study contributes to the evolving field of AI-ECG diagnostics by integrating both transparency and robustness assessments into model validation. Unlike previous work focused on performance alone, we aimed to provide a more holistic evaluation framework aligned with clinical needs. The main findings of our research can be summarized as follows: Firstly, the AI-ECG model exhibited substantial diagnostic precision for LVSD, achieving an AUROC of 0.919 (95% CI 0.887–0.951) [12]. Secondly, TCAVs and clustering analyses revealed that the model primarily relied on well-established and visually recognizable ECG features, such as LBBB and AF, which have known associations with LVSD. This finding supports the model’s alignment with clinical knowledge and enhances its transparency. Thirdly, robustness evaluations, encompassing lead reversal and noise tests, demonstrated the model’s steadfast performance in diverse clinical settings, underlining its adaptability and dependability for practical use. This comprehensive approach advances our understanding of AI-ECG’s potential in cardiac care and sets a new benchmark for integrating transparency and robustness in medical diagnostics.

The AI-ECG model demonstrated robust LVSD detection, evidenced by an impressive AUROC of 0.919 for binary outputs and 0.971 for continuous outputs. This validation approach, favoring clinical applicability, introduces binary cutoffs that resonate with healthcare professionals’ operational needs [32]. Although the 9.7 threshold was derived from prior development studies to ensure 90% sensitivity, we evaluated its diagnostic performance in this cohort, as shown in Supplementary Table S3. While it was not re-optimized for the current dataset, the threshold retained consistent performance characteristics and was used to maintain operational consistency for potential clinical deployment. Future studies may explore data-driven threshold optimization tailored to specific populations. The model effectively identified LV dysfunction below an LVEF of 40%, with higher AiTiALVSD scores indicating increased LVSD likelihood. Scores exceeding 53.5 were uniquely indicative of LVSD, while scores around the 9.7 cutoff were associated with mild LVEF reduction, notably with over half of the false positives showing an LVEF in the 40–50% range. This nuanced analysis highlights the model’s diagnostic acumen and clinical utility. However, it is important to interpret these findings in light of the study’s limitations. Although the model demonstrated high diagnostic accuracy, we acknowledge that this may reflect potential overestimation due to the absence of large-sized external validation. Further studies in independent, multi-institutional cohorts are needed to confirm these findings.

In a subgroup analysis of 96 patients with available NT-proBNP data, AiTiALVSD demonstrated a sensitivity of 92.6%, compared to 88.9% for NT-proBNP. Although this difference did not reach statistical significance due to overlapping confidence intervals, the results suggest that AI-ECG may offer comparable diagnostic performance and could warrant further investigation as a complementary tool for LVSD screening [4,20,21].

Traditional AI-ECG analysis, predominantly using SHapley Additive exPlanations (SHAP) for basic feature analysis or visual tools like GradCAM for localized insights, often struggles with the depth of explainability required for clinical application, particularly with deep learning models handling extensive datasets [33,34]. Such methods offer limited clinical insight, underscoring a need for a more holistic interpretative approach [35].

Addressing this gap, our study integrates advanced methodologies such as TCAV and clustering analysis [24,25]. This strategy not only aligns the AI model’s functionality with established medical knowledge but also ensures global interpretability, which is crucial for practical medical use. Through TCAV, the model’s reliance on critical ECG concepts for LVSD prediction—like QRS prolongation and abnormal ECG findings—is elucidated, significantly improving transparency and overcoming the conventional black-box critique. In particular, high TCAV scores for concepts such as LBBB, AF, and abnormal T waves reflect the model’s alignment with clinical reasoning patterns commonly used by cardiologists in diagnosing LVSD. This suggests that AiTiALVSD not only detects patterns that are statistically relevant, but also mirrors features physicians would intuitively consider, thereby enhancing its interpretability and trustworthiness. Furthermore, our clustering analysis distinguished five unique LVSD-positive ECG clusters, demonstrating the syndrome’s variability and affirming the model’s capacity to grasp LVSD’s complexity in a manner consistent with medical understanding. These clusters, characterized by diverse ECG profiles such as QRS widening, ST-T abnormalities, or atrial arrhythmias, may reflect known clinical subtypes such as ischemic or non-ischemic cardiomyopathy, offering an additional layer of insight for patient stratification and future risk assessment. This level of detail, showcased through transparent, visual representation, validates AiTiALVSD’s ability to navigate LVSD’s nuances, reinforcing the model’s clinical relevance and trustworthiness [20]. Transparency in the model’s development process, as detailed in Supplementary Text S2, further establishes a foundation for trust in AI-ECG diagnostics. While this study focused on evaluating global interpretability, we acknowledge that real-world clinical integration of explainability tools remains an important next step. Future directions include the development of clinician-facing interfaces that visualize concept-based insights, as well as the incorporation of local interpretability methods such as SHAP or Grad-CAM to support individualized decision-making at the point of care. While TCAVs and clustering analyses offer valuable insights into the model’s decision-making process, their clinical utility ultimately depends on how these insights are presented to end users. One possible direction is to convert concept-level attributions into simplified summary labels—such as “AF-like pattern detected” or “features suggestive of LBBB”—that align with familiar ECG terminology. Similarly, clustering results could be visualized as patient phenotype groups with descriptive ECG summaries (e.g., “ST-T abnormality dominant subtype”), enabling clinicians to interpret model outputs in a clinically intuitive manner. These approaches may bridge the gap between advanced model interpretation and real-world decision-making support.

Transitioning AI-ECG models like AiTiALVSD from research frameworks to clinical environments necessitates a thorough evaluation of their resilience against common practical challenges, including inaccuracies in lead placement and environmental noise. Our extensive robustness assessment of AiTiALVSD through lead reversal simulations in a 12-lead ECG configuration and exposure to diverse noise conditions showcased the model’s remarkable resilience. AiTiALVSD achieved an AUROC of 0.893 to 0.971 and sensitivity rates from 80.6% to 92.9% across lead reversal conditions, and an AUROC of 0.950 to 0.975 with sensitivities ranging from 81.2% to 92.1% amidst various noise types. These results underscore AiTiALVSD’s steady and reliable diagnostic capabilities in adverse settings. Such comprehensive evaluation not only underscores AiTiALVSD’s clinical deployment readiness but also establishes a new robustness benchmark for AI-ECG models, paving the way for future advancements in the discipline.

Our study’s insights come with inherent constraints that necessitate cautious interpretation. Its retrospective design and single-center focus within the Republic of Korea may limit the generalizability of the findings, particularly given the lack of global population diversity and real-time clinical decision-making. Although the dataset was generated under controlled conditions with clear criteria, potential selection and information biases cannot be entirely excluded. To address these limitations, future studies are planned to include external validation using datasets from different institutions and geographic regions. Prospective, multi-center investigations will be essential to confirm the model’s performance across more diverse and representative clinical settings. Furthermore, the absence of longitudinal follow-up in the current study restricts our ability to assess the model’s predictive value over time. Future research should therefore explore whether AiTiALVSD can serve not only as a diagnostic tool but also as a digital biomarker for monitoring disease progression and predicting long-term clinical outcomes. Additionally, while the 688 patients in our study were randomly selected from a total cohort of 7834 patients, this selection process may have introduced a potential bias. Although the dataset was generated under a controlled environment with clear criteria, the possibility of bias cannot be fully excluded. Furthermore, the study cohort may lack sufficient demographic diversity in terms of ethnicity, age, and comorbid conditions. Future validation in more diverse populations is essential to confirm the model’s performance across underrepresented subgroups. In particular, some subgroups with smaller sample sizes such as atrial fibrillation and chronic kidney disease showed wider confidence intervals in AUROC estimates, which may affect statistical certainty. This highlights the need for larger validation cohorts to strengthen subgroup-level inferences. Moreover, while the model displayed 80–90% sensitivity at a 9.7 binary cutoff, this threshold’s universality across diverse cohorts remains uncertain. Although the ECG–echocardiography interval differed slightly between groups, all ECGs were acquired within 24 h, a timeframe commonly accepted in prior AI-ECG studies [5,6,7,8]. We therefore consider the clinical impact minimal, although we acknowledge this as a potential limitation. The model’s adaptability, akin to traditional biomarkers, underscores the necessity for ongoing validation in varied clinical settings to affirm its reliability and broader clinical utility. Although this study applied TCAV to explore global interpretability, we acknowledge that it does not provide local, patient-level explanations. Future extensions of this work will incorporate local interpretability techniques such as SHAP or Grad-CAM, which are essential for supporting individualized clinical decisions and enhancing trust in AI-ECG diagnostics [36]. As an initial step toward local interpretability, we included representative Grad-CAM visualizations for two patient cases, one with a high AiTiALVSD score (true positive) and one with a low score (true negative), presented in Supplementary Figures S10 and S11. These examples illustrate how the model focuses on clinically relevant ECG segments associated with LVSD or its absence, providing intuitive insight into the regions of the input that influence the model’s prediction for individual cases.

Another practical limitation arises from the model’s decreased specificity under certain types of ECG noise, particularly EMG interference. While the model generally performed well under various noise conditions, this decline in performance suggests the need for targeted improvement strategies. One potential approach is to incorporate EMG-contaminated signals during model training through data augmentation, enabling the model to learn noise-resilient patterns. In parallel, advanced preprocessing techniques—such as adaptive filtering or deep learning-based denoising—may help reduce EMG artifacts prior to model inference. Together, these strategies could enhance both the robustness and reliability of the model in real-world clinical environments.

Future research should include larger sample sizes and longitudinal studies to explore specific prognostic indicators and refine the clinical utility of the findings. Furthermore, we plan to explore strategies to mitigate the impact of class imbalance by applying techniques such as oversampling, cost-sensitive learning, or resampling during model training and validation. In addition, the use of LVEF alone as the reference standard may not fully capture the pathophysiological spectrum of systolic dysfunction. Incorporating complementary modalities such as myocardial strain imaging or cardiac biomarkers could enhance the clinical validity of the AI-ECG model.

## 5. Conclusions

In conclusion, this study confirms the effectiveness of our AI-ECG model in detecting LVSD, emphasizing its accuracy, transparency, and robustness. It suggests the importance of using TCAV for comprehensive interpretation and conducting noise and lead reversal resilience tests. This comprehensive approach advances our understanding of AI-ECG’s potential in cardiac care and establishes a methodological benchmark for evaluating and integrating transparency and robustness in diagnostics.

## Figures and Tables

**Table 1:** Baseline characteristics.

	LVSD *n* = 98	Non-LVSD *n* = 583	*p* Value
Age	67.5 ± 13.6	59.6 ± 14.7	<0.001
Male	67 (68.4)	283 (48.5)	<0.001
Height	164.7 ± 9.3	163.6 ± 9.3	0.299
Weight	66.8 ± 13.8	66.8 ± 13.4	0.963
Medical History			
Hypertension	48 (49.0)	240 (41.2)	0.147
Diabetes mellitus	48 (49.0)	93 (16.0)	<0.001
Coronary artery disease	36 (36.7)	68 (11.7)	<0.001
Chronic kidney disease	14 (14.3)	17 (2.9)	<0.001
LVEF, %	33.2 ± 6.3	64.0 ± 6.9	<0.001
Echo-ECG time difference, hours	1.5 ± 3.6	3.0 ± 5.9	0.02
AiTiALVSD score	46.3 ± 28.2	2.6 ± 6.6	<0.001

Values are expressed as *n* (%), mean ± standard deviation. LVEF, left ventricular ejection fraction; Echo, echocardiography; ECG, electrocardiography; LVSD, left ventricular systolic dysfunction.

**Table 2:** AiTiALVSD classification performance.

	^a^ AUROC (95% CI)	^b^ AUROC (95% CI)	Sensitivity (95% CI)	Specificity (95% CI)	PPV (95% CI)	NPV (95% CI)
AiTiALVSD	0.919	0.971	0.898	0.940	0.715	0.982
	(0.887–0.951)	(0.954–0.989)	(0.838–0.958)	(0.921–0.959)	(0.636–0.795)	(0.971–0.993)

^a^ AUROC with binary output. ^b^ AUROC with continuous output. AUROC, area under the receiver operating characteristic curve; PPV, positive predictive value; NPV, negative predictive value; CI, confidence interval.

## Data Availability

Individual participant data generated by this study, after deidentification, will be made available upon reasonable request.

## Supplementary Materials

The following supporting information can be downloaded at: https://www.mdpi.com/article/10.3390/diagnostics15151837/s1, Supplementary Figure S1. An example of ECG noise type. Supplementary Figure S2. AiTiALVSD score distribution. Supplementary Figure S3. Pearson’s correlation analysis using the AiTiALVSD score and LVEF. Supplementary Figure S4. Forest plots of subgroup analyses. Supplementary Figures S5–S9. An example ECG of cluster A, B, C, D, and E. Supplementary Figure S10. Representative Grad-CAM visualization for a true positive case with a high AiTiALVSD score. Supplementary Figure S11. Representative Grad-CAM visualization for a true negative case with a low AiTiALVSD score. Supplementary Table S1. Tertiary outcomes. Supplementary Table S2. AiTiALVSD performance by subgroup. Supplementary Table S3. The performance of AiTiALVSD using a continuous output variable. Supplementary Table S4. Performance comparison between NT-proBNP and AiTiALVSD among 96 patients. Supplementary Table S5. Phenotyping of AiTiALVSD-positive LVSD cases. Supplementary Table S6. Diagnostic performance of AiTiALVSD: 12 types of lead reversal ECGs. Supplementary Table S7. Diagnostic performance of AiTiALVSD: 6 types of ECG noise. Supplementary Table S8. TCAV scores with 95% confidence intervals by feature block and concept. Supplementary Text S1. AiTiALVSD algorithm development. Supplementary Text S2. Model Card of AiTiALVSD for model development. Supplementary Text S3. Interpretation methods for model transparency. Supplementary Text S4. Sample size calculation.

## References

[1] McDonagh T.A., Metra M., Adamo M., Gardner R.S., Baumbach A., Böhm M., Burri H., Butler J., Čelutkienė J., Chioncel O. (2022). 2021 ESC Guidelines for the diagnosis and treatment of acute and chronic heart failure. Eur. J. Heart Fail..

[2] Conrad N., Judge A., Tran J., Mohseni H., Hedgecott D., Crespillo A.P., Allison M., Hemingway H., Cleland J.G., McMurray J.J.V. (2018). Temporal trends and patterns in heart failure incidence: A population-based study of 4 million individuals. Lancet.

[3] Heiat A., Gross C.P., Krumholz H.M. (2002). Representation of the elderly, women, and minorities in heart failure clinical trials. Arch. Intern. Med..

[4] Booth R.A., Hill S.A., Don-Wauchope A., Santaguida P.L., Oremus M., McKelvie R., Balion C., Brown J.A., Ali U., Bustamam A. (2014). Performance of BNP and NT-proBNP for diagnosis of heart failure in primary care patients: A systematic review. Heart Fail. Rev..

[5] Yao X., Rushlow D.R., Inselman J.W., McCoy R.G., Thacher T.D., Behnken E.M., Bernard M.E., Rosas S.L., Akfaly A., Misra A. (2021). Artificial intelligence–enabled electrocardiograms for identification of patients with low ejection fraction: A pragmatic, randomized clinical trial. Nat. Med..

[6] Sangha V., Nargesi A.A., Dhingra L.S., Khunte A., Mortazavi B.J., Ribeiro A.H., Banina E., Adeola O., Garg N., Brandt C.A. (2023). Detection of Left Ventricular Systolic Dysfunction from Electrocardiographic Images. medRxiv.

[7] Dhingra L.S., Aminorroaya A., Sangha V., Pedroso A.F., Asselbergs F.W., Brant L.C.C., Barreto S.M., Ribeiro A.L.P., Krumholz H.M., Oikonomou E.K. (2025). Heart failure risk stratification using artificial intelligence applied to electrocardiogram images: A multinational study. Eur. Heart J..

[8] Attia Z.I., Kapa S., Lopez-Jimenez F., McKie P.M., Ladewig D.J., Satam G., Pellikka P.A., Enriquez-Sarano M., Noseworthy P.A., Munger T.M. (2019). Screening for cardiac contractile dysfunction using an artificial intelligence–enabled electrocardiogram. Nat. Med..

[9] Kwon J., Kim K.-H., Jeon K.-H., Kim H.M., Kim M.J., Lim S.-M., Song P.S., Park J., Choi R.K., Oh B.-H. (2018). Development and Validation of Deep-Learning Algorithm for Electrocardiography-Based Heart Failure Identification. Korean Circ. J..

[10] Kwon J., Jo Y.-Y., Lee S.Y., Kang S., Lim S.-Y., Lee M.S., Kim K.-H. (2022). Artificial Intelligence-Enhanced Smartwatch ECG for Heart Failure-Reduced Ejection Fraction Detection by Generating 12-Lead ECG. Diagnostics.

[11] Lee Y., Choi B., Lee M.S., Jin U., Yoon S., Jo Y.-Y., Kwon J. (2022). An artificial intelligence electrocardiogram analysis for detecting cardiomyopathy in the peripartum period. Int. J. Cardiol..

[12] Jung Y.M., Kang S., Son J.M., Lee H.S., Han G.I., Yoo A.-H., Kwon J., Park C.-W., Park J.S., Jun J.K. (2023). Electrocardiogram-based deep learning model to screen peripartum cardiomyopathy. Am. J. Obstet. Gynecol. MFM.

[13] Jeong J.H., Kang S., Lee H.S., Lee M.S., Son J.M., Kwon J., Lee H.S., Choi Y.Y., Kim S.R., Cho D.-H. (2024). Deep learning algorithm for predicting left ventricular systolic dysfunction in atrial fibrillation with rapid ventricular response. Eur. Heart J.-Digit. Health.

[14] Stower H. (2020). Transparency in medical AI. Nat. Med..

[15] Shick A.A., Webber C.M., Kiarashi N., Weinberg J.P., Deoras A., Petrick N., Saha A., Diamond M.C. (2024). Transparency of artificial intelligence/machine learning-enabled medical devices. Npj Digit. Med..

[16] Jain S.S., Elias P., Poterucha T., Randazzo M., Jimenez F.L., Khera R., Perez M., Ouyang D., Pirruccello J., Salerno M. (2024). Artificial Intelligence in Cardiovascular Care—Part 2: Applications JACC Review Topic of the Week. J. Am. Coll. Cardiol..

[17] Jiang J., Jiang X., Xu L., Zhang Y., Zheng Y., Kong D. (2023). Noise-robustness test for ultrasound breast nodule neural network models as medical devices. Front. Oncol..

[18] Shen C., Tsai M.-Y., Chen L., Li S., Nguyen D., Wang J., Jiang S.B., Jia X. (2020). On the robustness of deep learning-based lung-nodule classification for CT images with respect to image noise. Phys. Med. Biol..

[19] Al-Zaiti S.S., Bond R.R. (2023). Explainable-by-design: Challenges, pitfalls, and opportunities for the clinical adoption of AI-enabled ECG. J. Electrocardiol..

[20] McDonagh T., Robb S., Murdoch D., Morton J., Ford I., Morrison C., Tunstall-Pedoe H., McMurray J., Dargie H. (1998). Biochemical detection of left-ventricular systolic dysfunction. Lancet.

[21] Bay M., Kirk V., Parner J., Hassager C., Nielsen H., Krogsgaard K., Trawinski J., Boesgaard S., Aldershvile J. (2003). NT-proBNP: A new diagnostic screening tool to differentiate between patients with normal and reduced left ventricular systolic function. Heart.

[22] He K., Zhang X., Ren S., Sun J. (2015). Deep Residual Learning for Image Recognition. arXiv.

[23] Paszke A., Gross S., Massa F., Lerer A., Bradbury J., Chanan G., Killeen T., Lin Z., Gimelshein N., Antiga L. (2019). PyTorch: An Imperative Style, High-Performance Deep Learning Library. arXiv.

[24] Bienefeld N., Boss J.M., Lüthy R., Brodbeck D., Azzati J., Blaser M., Willms J., Keller E. (2023). Solving the explainable AI conundrum by bridging clinicians’ needs and developers’ goals. Npj Digit. Med..

[25] Kim B., Wattenberg M., Gilmer J., Cai C., Wexler J., Viegas F., Sayres R. (2017). Interpretability Beyond Feature Attribution: Quantitative Testing with Concept Activation Vectors (TCAV). arXiv.

[26] Alday E.A.P., Gu A., Shah A.J., Robichaux C., Wong A.-K.I., Liu C., Liu F., Rad A.B., Elola A., Seyedi S. (2020). Classification of 12-lead ECGs: The PhysioNet/Computing in Cardiology Challenge 2020. Physiol. Meas..

[27] O’Neal W.T., Mazur M., Bertoni A.G., Bluemke D.A., Al-Mallah M.H., Lima J.A.C., Kitzman D., Soliman E.Z. (2017). Electrocardiographic Predictors of Heart Failure With Reduced Versus Preserved Ejection Fraction: The Multi-Ethnic Study of Atherosclerosis. J. Am. Heart Assoc..

[28] Olesen L.L., Andersen A. (2016). ECG as a first step in the detection of left ventricular systolic dysfunction in the elderly. ESC Heart Fail..

[29] Kokhlikyan N., Miglani V., Martin M., Wang E., Alsallakh B., Reynolds J., Melnikov A., Kliushkina N., Araya C., Yan S. (2020). Captum: A unified and generic model interpretability library for PyTorch. arXiv.

[30] Satopää V., Albrecht J., Irwin D., Raghavan B. Finding a “Kneedle” in a Haystack: Detecting Knee Points in System Behavior. Proceedings of the 2011 31st International Conference on Distributed Computing Systems Workshops.

[31] Batchvarov V.N., Malik M., Camm A.J. (2007). Incorrect electrode cable connection during electrocardiographic recording. EP Eur..

[32] Ulloa-Cerna A.E., Jing L., Pfeifer J.M., Raghunath S., Ruhl J.A., Rocha D.B., Leader J.B., Zimmerman N., Lee G., Steinhubl S.R. (2022). rECHOmmend: An ECG-Based Machine Learning Approach for Identifying Patients at Increased Risk of Undiagnosed Structural Heart Disease Detectable by Echocardiography. Circulation.

[33] Selvaraju R.R., Cogswell M., Das A., Vedantam R., Parikh D., Batra D. Grad-CAM: Visual Explanations from Deep Networks Via Gradient-Based Localization. Proceedings of the 2017 IEEE International Conference on Computer Vision (ICCV).

[34] Lundberg S., Lee S.-I. (2017). A Unified Approach to Interpreting Model Predictions. arXiv.

[35] Gliner V., Levy I., Tsutsui K., Acha M.R., Schliamser J., Schuster A., Yaniv Y. (2025). Clinically meaningful interpretability of an AI model for ECG classification. Npj Digit. Med..

[36] Oikonomou E.K., Khera R. (2024). Artificial intelligence-enhanced patient evaluation: Bridging art and science. Eur. Heart J..

