# Supplementary material for: Transparent and Robust Artificial Intelligence-Driven Electrocardiogram Model for Left Ventricular Systolic Dysfunction
Source: Diagnostics (Basel). 2025 Jul 22;15(15):1837. doi: 10.3390/diagnostics15151837 (PMC12346549; doi:10.3390/diagnostics15151837)
Supplement: Supplementary file 1 [file diagnostics-15-01837-s001.zip › diagnostics-3710561-supplementary.pdf]

Supplementary Content

**Supplementary Figure S1.**	An example of ECG noise type.
**Supplementary Figure S2.**	AiTiALVSD score distribution.
**Supplementary Figure S3.**	Pearson's correlation analysis using the AiTiALVSD score and LVEF
**Supplementary Figure S4.**	Forest plots of subgroup analyses
**Supplementary Figures S5–S9.**	An example ECG of cluster A, B, C, D and E.
**Supplementary Figure S10.**	Representative Grad-CAM visualization for a true positive case with a high AiTiALVSD score
**Supplementary Figure S11.**	Representative Grad-CAM visualization for a true negative case with a low AiTiALVSD score
**Supplementary Table S1.**	Tertiary outcomes
**Supplementary Table S2.**	AiTiALVSD performance by subgroup
**Supplementary Table S3.**	The performance of AiTiALVSD using a continuous output variable
**Supplementary Table S4.**	Performance comparison between NT-proBNP and AiTiALVSD among 96 patients
**Supplementary Table S5.**	Phenotyping of AiTiALVSD-positive LVSD cases
**Supplementary Table S6.**	Diagnostic performance of AiTiALVSD; 12 types of lead reversal ECGs
**Supplementary Table S7.**	Diagnostic performance of AiTiALVSD; 6 types of ECG noise
**Supplementary Table S8.**	TCAV scores with 95% confidence intervals by feature block and concept
**Supplementary Text S1.**	AiTiALVSD algorithm development
**Supplementary Text S2.**	Model Card of AiTiALVSD for model development
**Supplementary Text S3.**	Interpretation methods for model transparency
**Supplementary Text S4.**	Sample size calculation

Supplementary Figures

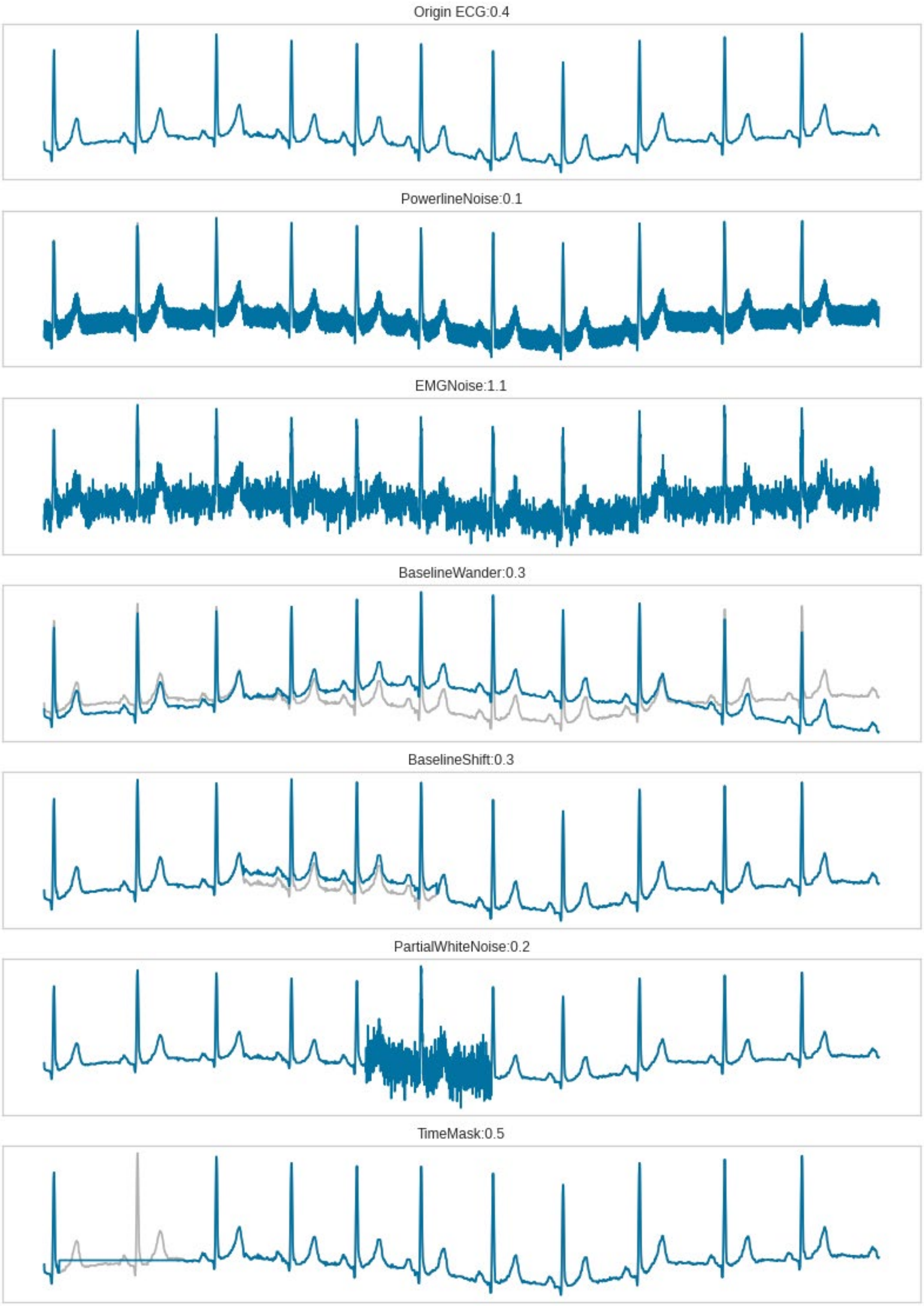

Supplementary Figure S1. An example of ECG noise type

Each score is the AiTiALVSD score when augmented ECG (blue) with each type of noise is predicted. Gray-colored ECG is an original ECG.

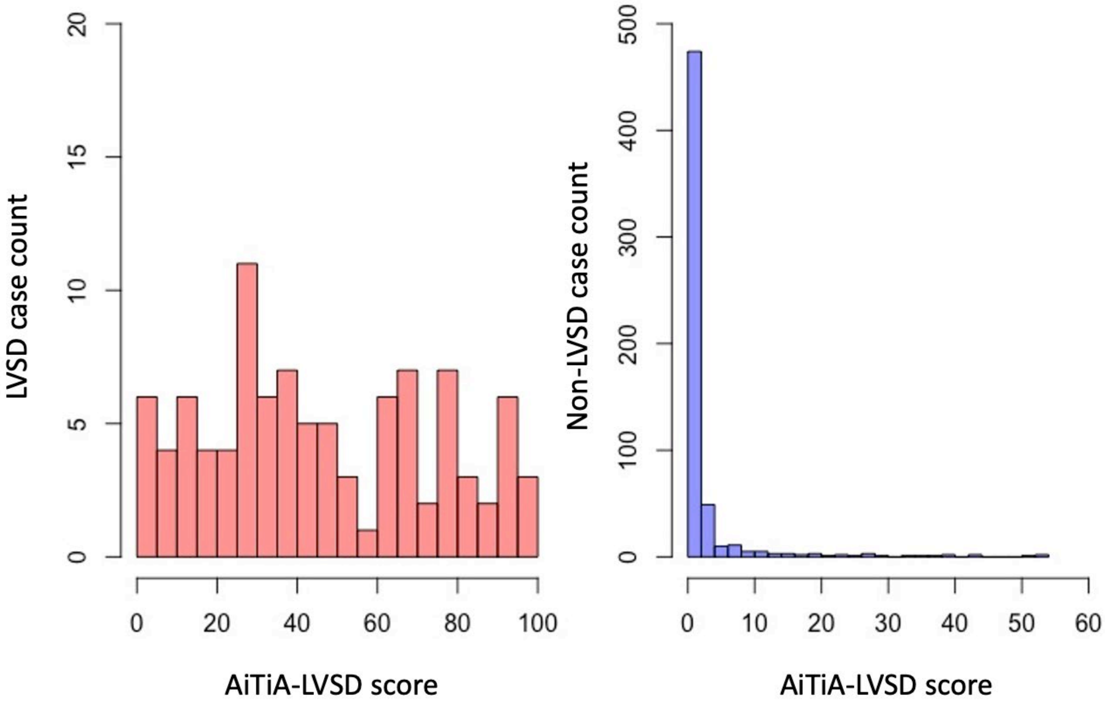

Supplementary Figure S2. AiTiALVSD score distribution.

When the score distribution in the LVSD and Non-LVSD groups is expressed as a histogram, the two groups are clearly distributed with scores above and below 9.7

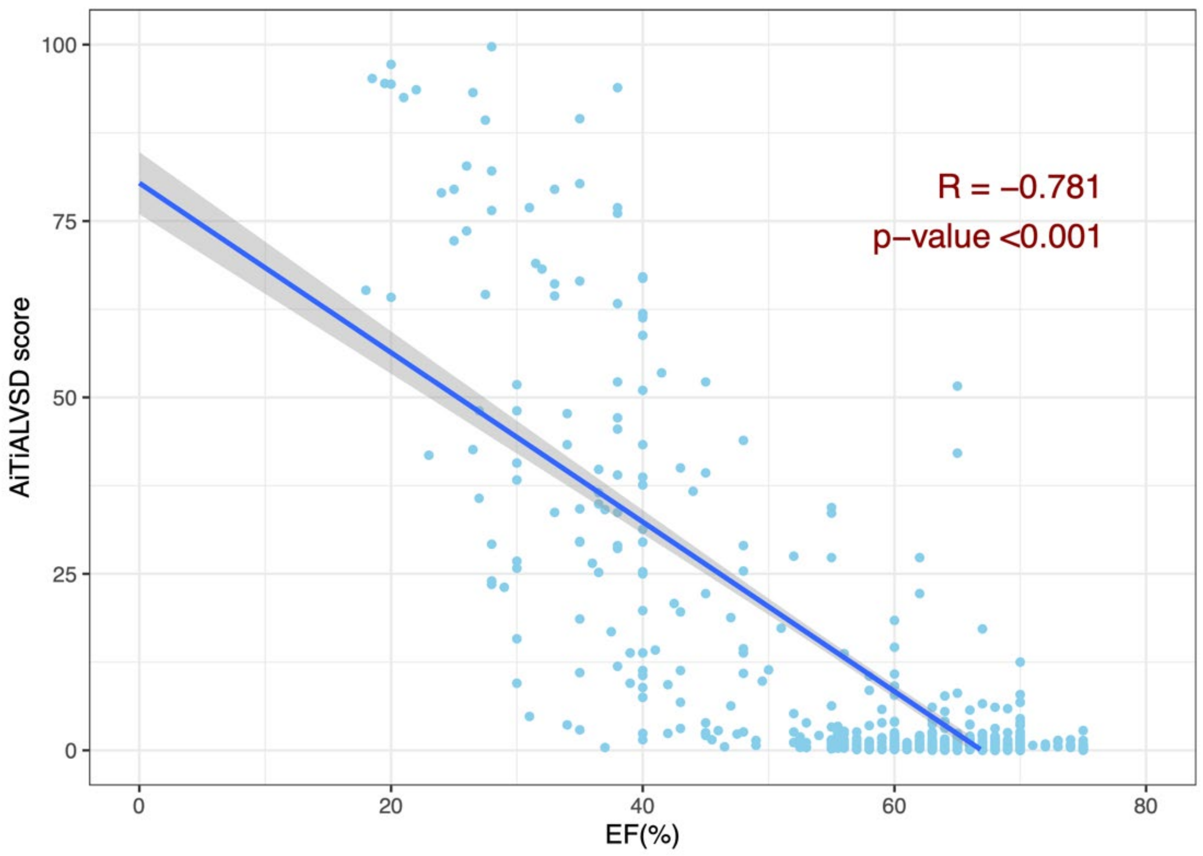

Supplementary Figure S3. Pearson's correlation analysis using the AiTiALVSD score and LVEF

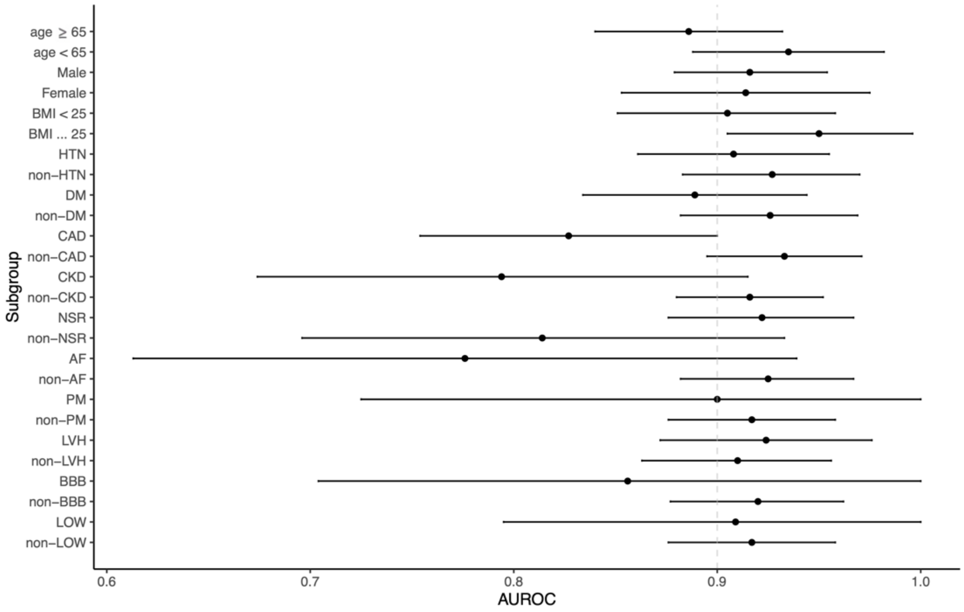

Supplementary Figure S4. Forest plots of subgroup analyses

The AUROC and 95% confidence interval of AiTiALVSD in subgroups according to baseline characteristics and ECG interpretation are displayed in a forest plot; it shows overall performance at 0.8 to 0.9. BMI, body mass index; HTN, hypertension; DM, diabetes mellitus; CAD, coronary artery disease; CKD, chronic kidney disease; SR, sinus rhythm; AF, atrial fibrillation; PM, pacemaker; LVH, left ventricular hypertrophy; BBB, right and left bundle branch block; LOW, low voltage

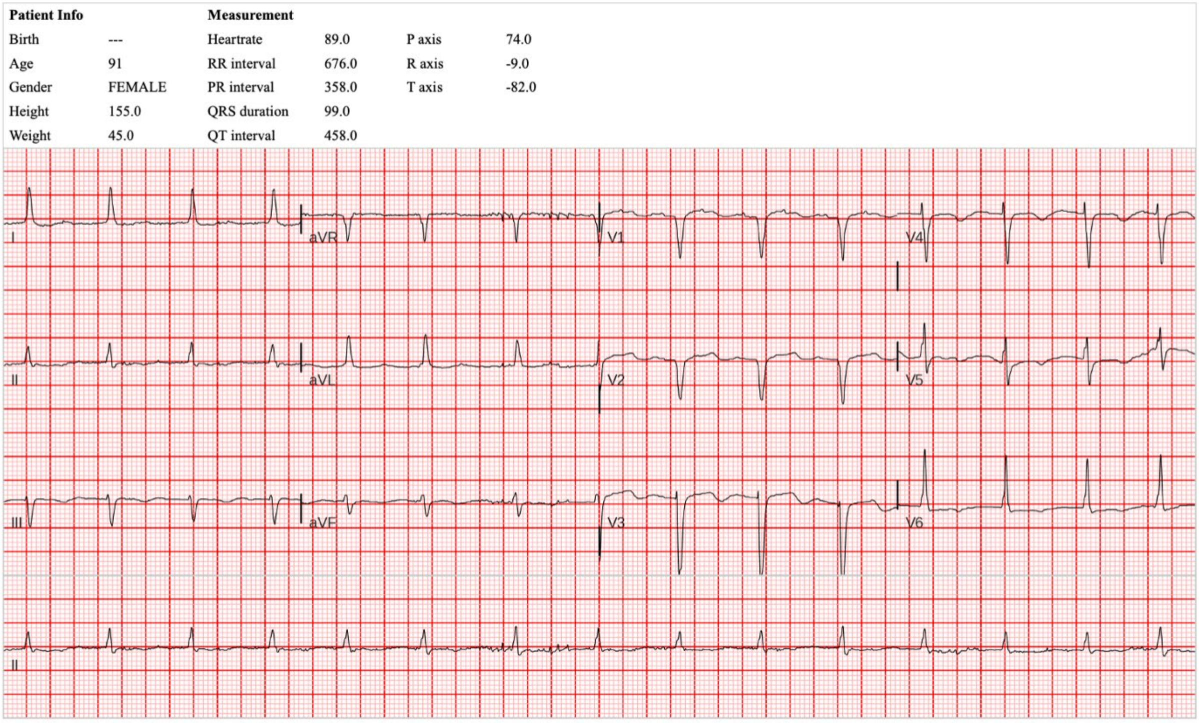

Supplementary Figure S5. An example ECG of cluster A

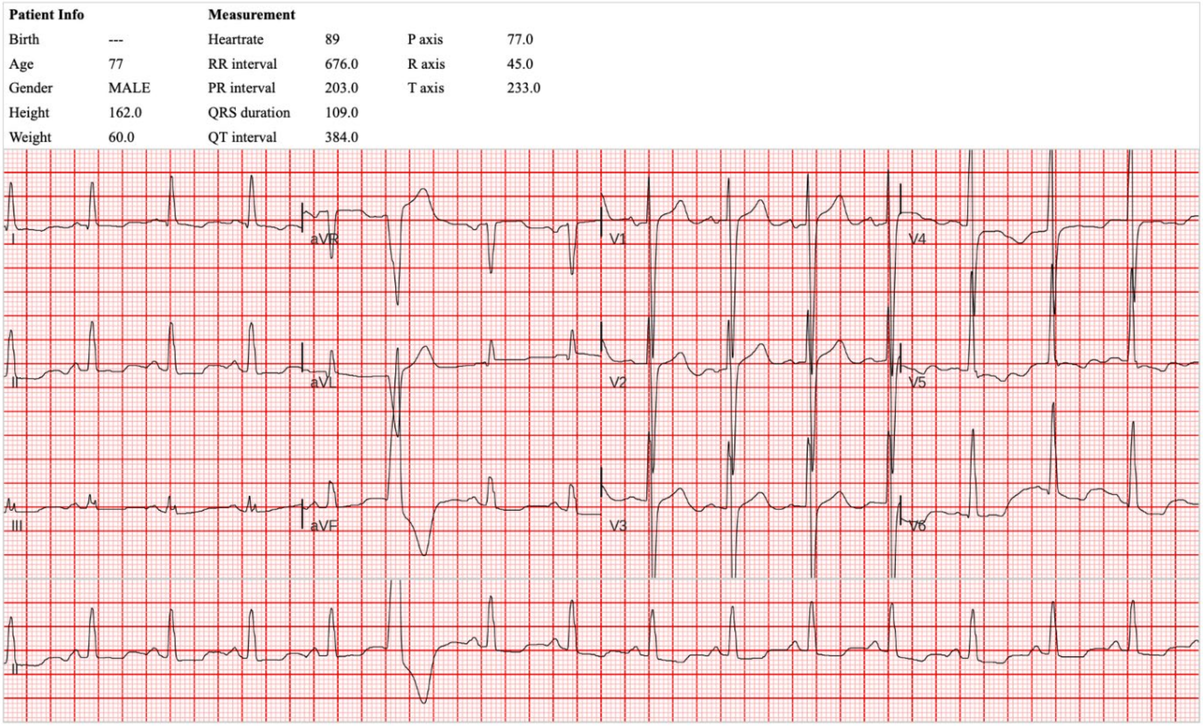

Supplementary Figure S6. An example ECG of cluster B

Patient Info		Measurement			
Birth	---	Heartrate	76.0	P axis	58.0
Age	81	RR interval	788.0	R axis	12.0
Gender	MALE	PR interval	158.0	T axis	-71.0
Height	168.0	QRS duration	106.0		
Weight	58.0	QT interval	458.0		

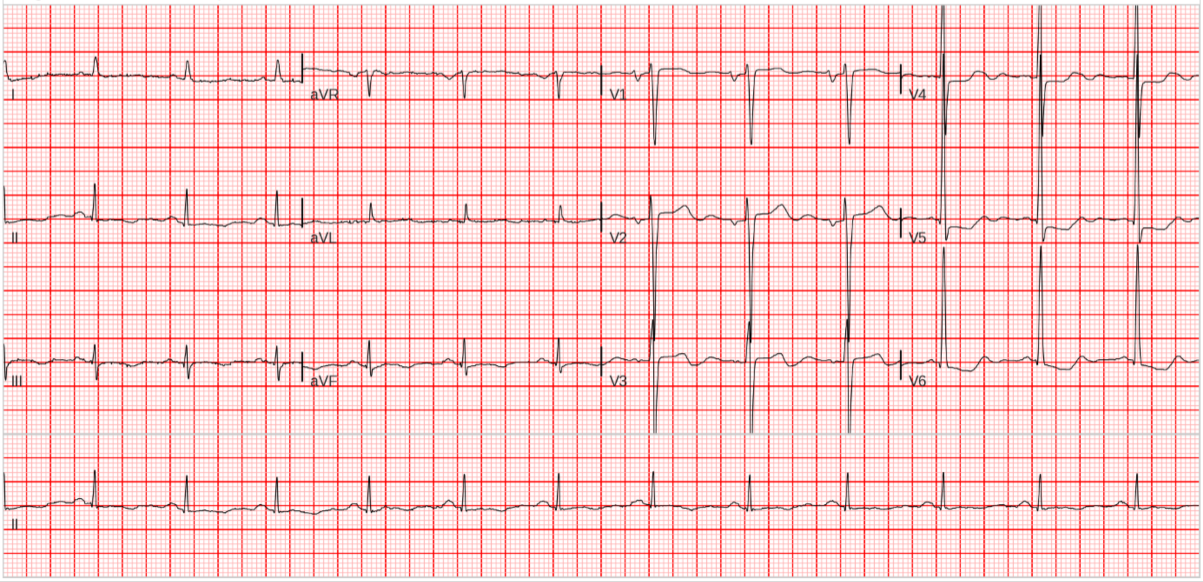

Supplementary Figure S7. An example ECG of cluster C

Patient Info		Measurement			
Birth	---	Heartrate	55.0	P axis	---
Age	75	RR interval	1092.0	R axis	-61.0
Gender	MALE	PR interval	---	T axis	96.0
Height	160.0	QRS duration	144		
Weight	73.0	QT interval	464		

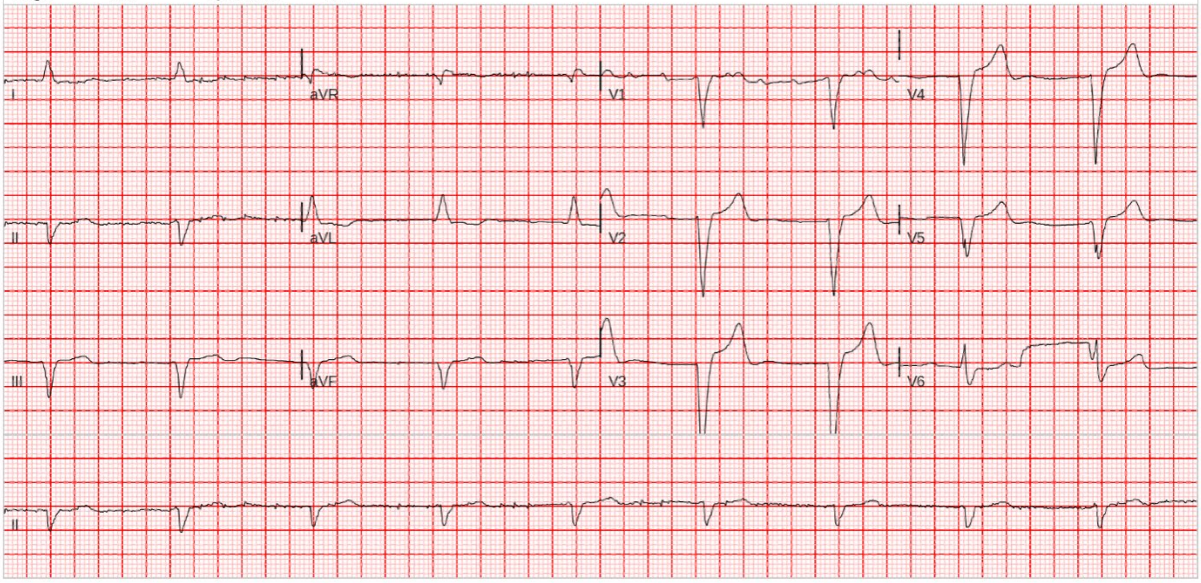

Supplementary Figure S8. An example ECG of cluster D

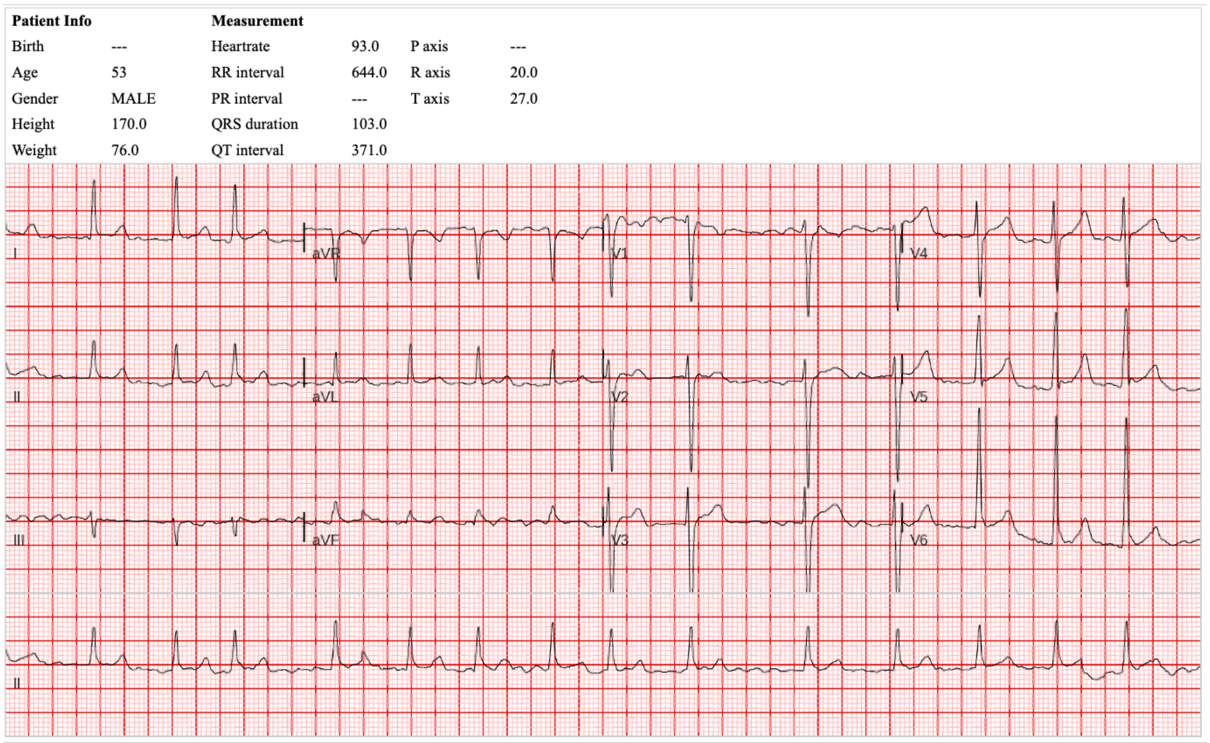

Supplementary Figure S9. An example ECG of cluster E

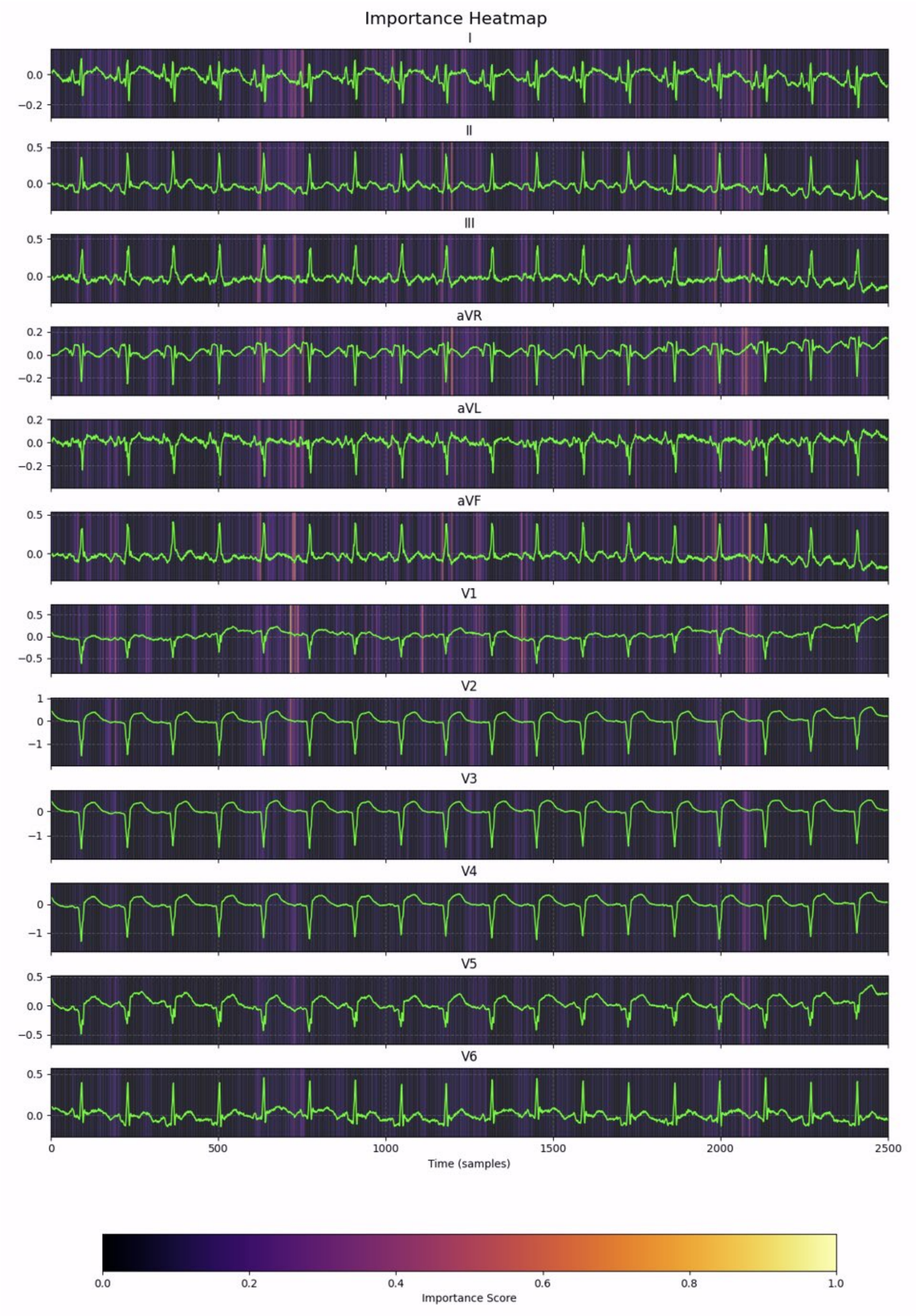

Supplementary Figure S10. Representative Grad-CAM visualization for a true positive case with a high AiTiALVSD score

Grad-CAM heatmap for a patient with LVSD and a high AiTiALVSD score (score = 70.1). The visualization highlights specific ECG segments contributing most to the model's prediction, corresponding to regions with p wave.

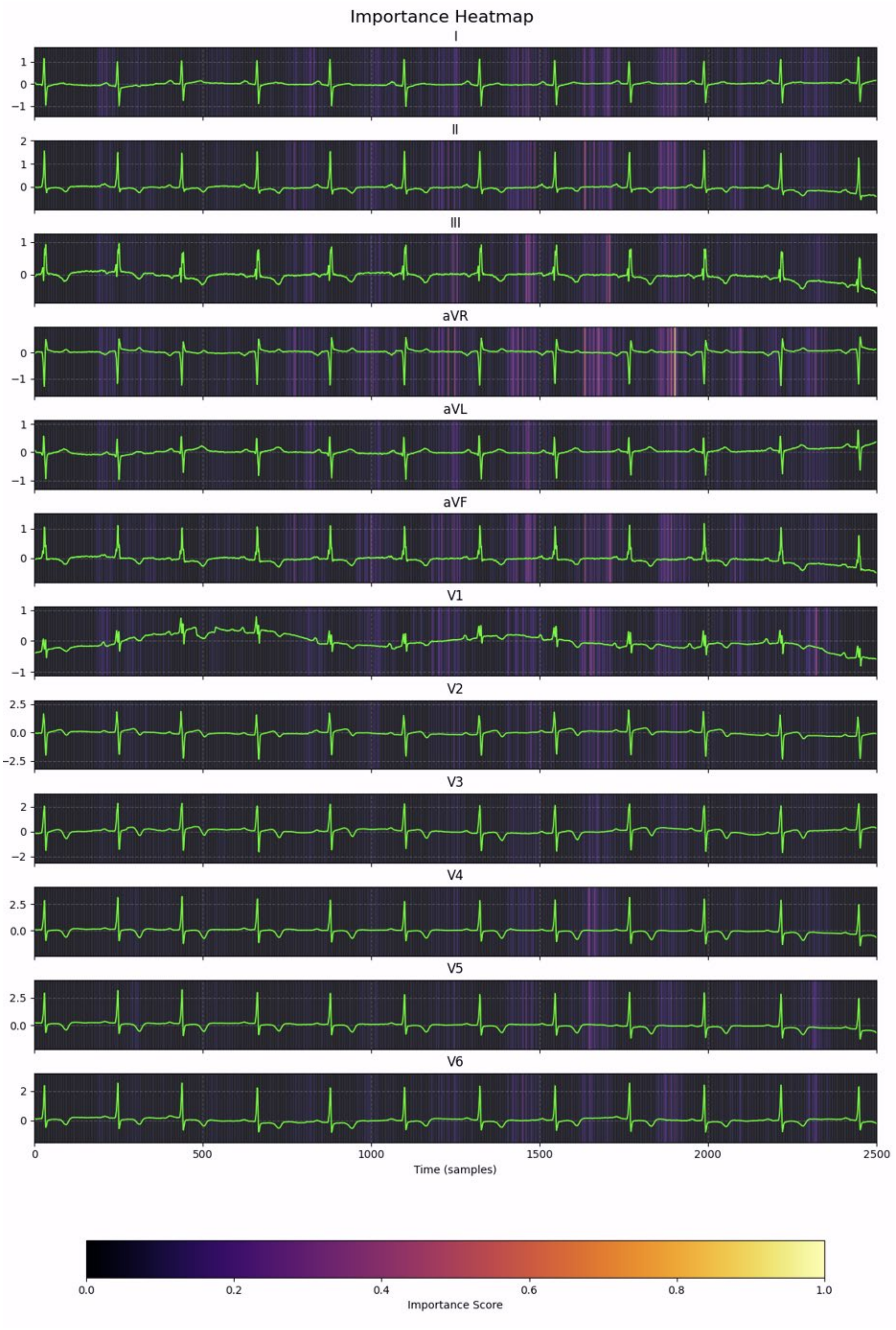

Supplementary Figure S11. Representative Grad-CAM visualization for a true negative case with a low AiTiALVSD score

Grad-CAM heatmap for a patient without LVSD and a low AiTiALVSD score (score = 2.8). The model assigns minimal attention to regions without notable abnormal patterns, consistent with a low LVSD probability.

Supplementary Tables

Supplementary Table S1. Tertiary outcomes

	AUROC (95% CI)	Sensitivity (95% CI)	Specificity (95% CI)	PPV (95% CI)	NPV (95% CI)
AiTiALVSD (LVEF ≤ 35%)	0.906 (0.869-0.943)	0.927 (0.859-0.996)	0.885 (0.860-0.910)	0.415 (0.328-0.502)	0.993 (0.986-1.0)
AiTiALVSD (LVEF ≤ 50%)	0.891 (0.857-0.925)	0.811 (0.744-0.877)	0.971 (0.957-0.985)	0.870 (0.810-0.929)	0.955 (0.938-0.972)

LVEF, left ventricular ejection fraction; AUROC, area under the receiver operating characteristic curve; PPV, positive predictive value; NPV, negative predictive value; CI, confidence interval

Supplementary Table S2. AiTiALVSD performance by subgroup

	AUROC (95% CI)	AUPRC (95% CI)	Sensitivity (95% CI)	Specificity (95% CI)	PPV (95% CI)	NPV (95% CI)
Age						
age ≥ 65 (n=263)	0.886 (0.840-0.932)	0.591 (0.497-0.683)	0.906 (0.827-0.984)	0.867 (0.821-0.913)	0.632 (0.523-0.740)	0.973 (0.950-0.996)
age < 65 (n=417)	0.935 (0.888-0.982)	0.768 (0.658-0.868)	0.889 (0.797-0.981)	0.981 (0.967-0.995)	0.851 (0.749-0.953)	0.986 (0.975-0.998)
Sex						
Male (n=350)	0.916 (0.879-0.954)	0.686 (0.605-0.764)	0.91 (0.842-0.979)	0.922 (0.891-0.953)	0.735 (0.640-0.830)	0.978 (0.960-0.995)
Female (n=331)	0.914 (0.853-0.975)	0.600 (0.460-0.736)	0.871 (0.753-0.989)	0.957 (0.934-0.980)	0.675 (0.530-0.820)	0.986 (0.973-1.0)
BMI						
BMI < 25 (n=396)	0.905 (0.851-0.958)	0.679 (0.588-0.759)	0.864 (0.781-0.946)	0.945 (0.921-0.970)	0.76 (0.663-0.857)	0.972 (0.954-0.99)
BMI ≥ 25 (n=282)	0.950 (0.905-0.996)	0.870 (0.780-0.940)	0.969 (0.908-1.00)	0.932 (0.901-0.963)	0.646 (0.511-0.781)	1.00 (0.987-1.00)
HTN						

HTN (n=288)	0.908 (0.861-0.955)	0.639 (0.541-0.742)	0.896 (0.809-0.982)	0.921 (0.887-0.955)	0.694 (0.579-0.808)	0.978 (0.959-0.997)
non-HTN (n=393)	0.927 (0.883-0.970)	0.677 (0.570-0.777)	0.9 (0.817-0.983)	0.953 (0.931-0.976)	0.738 (0.627-0.848)	0.985 (0.972-0.998)
DM						
DM (n=141)	0.889 (0.834-0.944)	0.749 (0.646-0.841)	0.896 (0.809-0.982)	0.88 (0.814-0.947)	0.796 (0.689-0.904)	0.942 (0.892-0.991)
non-DM (n=540)	0.926 (0.882-0.969)	0.596 (0.501-0.690)	0.9 (0.817-0.983)	0.951 (0.932-0.970)	0.652 (0.540-0.765)	0.989 (0.980-0.999)
CAD						
CAD (n=104)	0.827 (0.754-0.90)	0.631 (0.514-0.742)	0.889 (0.786-0.992)	0.765 (0.664-0.866)	0.667 (0.533-0.80)	0.929 (0.861-0.996)
non-CAD (n=577)	0.933 (0.895-0.971)	0.685 (0.583-0.771)	0.903 (0.830-0.977)	0.963 (0.947-0.979)	0.747 (0.648-0.845)	0.988 (0.979-0.998)
CKD						
CKD (n=31)	0.794 (0.674-0.915)	0.667 (0.478-0.826)	1.00 (1.00-1.00)	0.588 (0.354-0.822)	0.667 (0.465-0.868)	1.00 (1.00-1.00)
	0.916	0.655	0.881	0.951	0.725	0.982

non-CKD (n=650)	(0.880-0.952)	(0.577-0.733)	(0.812-0.950)	(0.933-0.968)	(0.639-0.812)	(0.971-0.993)
SR						
NSR (n=613)	0.922 (0.876-0.967)	0.659 (0.574-0.756)	0.886 (0.811-0.960)	0.958 (0.941-0.975)	0.729 (0.635-0.824)	0.985 (0.974-0.995)
non-NSR (n=68)	0.814 (0.696-0.933)	0.665 (0.542-0.794)	0.929 (0.833-1.00)	0.7 (0.558-0.842)	0.684 (0.536-0.832)	0.933 (0.844-1.00)
AF						
AF (n=43)	0.776 (0.613-0.939)	0.670 (0.526-0.818)	0.9 (0.769-1.00)	0.652 (0.458-0.847)	0.692 (0.515-0.870)	0.882 (0.729-1.00)
non-AF (n=638)	0.925 (0.882-0.967)	0.660 (0.580-0.739)	0.897 (0.830-0.965)	0.952 (0.934-0.970)	0.722 (0.632-0.811)	0.985 (0.975-0.995)
PM						
PM (n=10)	0.9 (0.725-1.00)	0.833 (0.500-1.00)	1.00 (1.00-1.00)	0.8 (0.449-1.00)	0.833 (0.535-1.00)	1.00 (1.00-1.00)
non-PM (n=671)	0.917 (0.876-0.958)	0.648 (0.571-0.723)	0.892 (0.830-0.955)	0.941 (0.922-0.960)	0.709 (0.627-0.792)	0.982 (0.971-0.993)
LVH						
	0.924	0.741	1.00	0.848	0.741	1.00

LVH (n=66)	(0.872-0.976)	(0.600-0.875)	(1.00-1.00)	(0.744-0.952)	(0.575-0.906)	(1.00-1.00)
non-LVH (n=615)	0.91 (0.863-0.956)	0.634 (0.546-0.712)	0.872 (0.798-0.946)	0.948 (0.929-0.967)	0.708 (0.617-0.799)	0.981 (0.969-0.993)
BBB						
BBB (n=37)	0.856 (0.704-1.00)	0.698 (0.503-0.867)	0.929 (0.794-1.00)	0.783 (0.614-0.951)	0.722 (0.515-0.929)	0.947 (0.847-1.00)
non-BBB (n=644)	0.92 (0.877-0.962)	0.652 (0.579-0.726)	0.893 (0.827-0.959)	0.946 (0.928-0.965)	0.714 (0.628-0.801)	0.983 (0.972-0.994)
LOW						
LOW (n=16)	0.909 (0.795-1.00)	0.714 (0.400-1.00)	1.00 (1.00-1.00)	0.818 (0.590-1.00)	0.714 (0.380-1.00)	1.00 (1.00-1.00)
non-LOW (n=665)	0.917 (0.876-0.958)	0.654 (0.580-0.723)	0.892 (0.830-0.955)	0.942 (0.923-0.961)	0.716 (0.633-0.798)	0.982 (0.971-0.993)

AUROC, area under the receiver operating characteristic curve; AUPRC, area under the precision-recall curve; PPV, positive predictive value; NPV, negative predictive value; CI, confidence interval; BMI, body mass index; HTN, hypertension; DM, diabetes mellitus; CAD, coronary artery disease; CKD, chronic kidney disease; SR, sinus rhythm; NSR, non-sinus

rhythm; AF, atrial fibrillation; PM, pacemaker; LVH, left ventricular hypertrophy; BBB, bundle branch block; LOW, low voltage.

Supplementary Table S3. The performance of AiTiALVSD using a continuous output variable

	AUROC (95% CI)	AUPRC (95% CI)
LVEF ≤ 40%	0.971 (0.956-0.985)	0.883 (0.839-0.925)
LVEF ≤ 35%	0.966 (0.951-0.980)	0.729 (0.622-0.819)
LVEF ≤ 50%	0.963 (0.948-0.982)	0.903 (0.867-0.935)
**Age**		
age ≥ 65	0.959	0.868
(n=263)	(0.936-0.982)	(0.806-0.925)
age < 65	0.974	0.918
(n=417)	(0.939-1.00)	(0.852-0.970)
**Sex**		
Male	0.961	0.890
(n=350)	(0.933-0.990)	(0.838-0.937)
Female	0.984	0.866
(n=331)	(0.972-0.996)	(0.779-0.938)
**BMI**		
BMI < 25	0.965	0.891
(n=396)	(0.940-0.991)	(0.839-0.935)
	0.980	0.837

25 ≤ BMI ≤ 30 (n=227)	(0.965-0.995)	(0.724-0.930)
BMI > 30	0.993	0.963
(n=58)	(0.978-1.00)	(0.885-1.00)
**HTN**		
HTN	0.967	0.863
(n=288)	(0.947-0.987)	(0.794-0.922)
non-HTN	0.972	0.907
(n=393)	(0.941-1.00)	(0.840-0.957)
**DM**		
DM	0.943	0.914
(n=141)	(0.901-0.985)	(0.861-0.958)
non-DM	0.983	0.865
(n=540)	(0.971-0.994)	(0.791-0.922)
**CAD**		
CAD	0.930	0.879
(n=104)	(0.882-0.977)	(0.797-0.944)
non-CAD	0.975	0.888
(n=577)	(0.950-1.00)	(0.827-0.939)
**CKD**		
CKD	0.933	0.934
(n=31)	(0.850-1.00)	(0.844-0.991)
non-CKD	0.971	0.875
(n=650)	(0.951-0.991)	(0.820-0.921)

**SR**		
NSR	0.972	0.877
(n=613)	(0.950-0.995)	(0.824-0.292)
non-NSR	0.915	0.893
(n=68)	(0.851-0.979)	(0.808-0.958)
**AF**		
AF	0.876	0.865
(n=43)	(0.775-0.977)	(0.756-0.956)
non-AF	0.973	0.886
(n=638)	(0.953-0.994)	(0.836-0.932)
**PM**		
PM	1.00	1.00
(n=10)	(1.00-1.00)	(1.00-1.00)
non-PM	0.970	0.875
(n=671)	(0.952-0.989)	(0.823-0.919)
**LVH**		
LVH	0.976	0.947
(n=66)	(0.948-1.00)	(0.883-0.990)
non-LVH	0.969	0.868
(n=615)	(0.947-0.991)	(0.812-0.914)
**BBB**		
BBB	0.960	0.943
(n=37)	(0.907-1.00)	(0.853-0.993)
	0.971	0.873

non-BBB (n=644)	(0.950-0.991)	(0.825-0.920)
**LOW**		
LOW	0.945	0.877
(n=16)	(0.830-1.00)	(0.600-1.00)
non-LOW	0.971	0.881
(n=665)	(0.953-0.990)	(0.837-0.921)

AUROC, area under the receiver operating characteristic curve; AUPRC, area under the precision-recall curve; CI, confidence interval; BMI, body mass index; HTN, hypertension; DM, diabetes mellitus; CAD, coronary artery disease; CKD, chronic kidney disease; SR, sinus rhythm; NSR, non-sinus rhythm; AF, atrial fibrillation; PM, pacemaker; LVH, left ventricular hypertrophy; BBB, bundle branch block; LOW, low voltage.

Supplementary Table S4. Performance comparison between NT-proBNP and AiTiALVSD among 96 patients

	AUROC (95% CI)	Sensitivity (95% CI)	Specificity (95% CI)	PPV (95% CI)	NPV (95% CI)	TN	TP	FN	FP
NT-proBNP	0.72 (0.635-0.804)	0.889 (0.770-1.0)	0.551 (0.433-0.668)	0.436 (0.305-0.567)	0.927 (0.847-1.0)	38	24	3	31
AiTiALVSD	0.905 (0.842-0.968)	0.926 (0.827-1.0)	0.884 (0.809-0.960)	0.758 (0.611-0.904)	0.968 (0.925-1.0)	61	25	2	8

AUROC, area under the receiver operating characteristic curve; PPV, positive predictive value; NPV, negative predictive value; CI, confidence interval; TN, true negative; TP, true positive; FN, false negative; FP, false positive.

Supplementary Table S5. Phenotyping of AiTiALVSD-positive LVSD cases

	Phenotype A	Phenotype B	Phenotype C	Phenotype D	Phenotype E	Total
	(n=16)	(n=13)	(n=32)	(n=49)	(n=13)	(n=123)
Age, year	66.25 ± 11.87	69.77 ± 11.30	69.22 ± 14.37	69.90 ± 14.06	68.15 ± 14.06	69.05 ± 13.45
Male, n (%)	13 (81.25)	9 (69.23)	20 (62.5)	34 (69.39)	7 (53.85)	83 (67.48)
Height, cm	166.19 ± 8.00	165.22 ± 8.42	162.86 ± 10.08	163.86 ± 9.95	164.17 ± 12.01	164.08 ± 9.74
Weight, kg	71.79 ± 16.42	74.85 ± 13.13	62.52 ± 11.84	66.82 ± 15.70	67.81 ± 19.56	67.30 ± 15.34
**Medical History**						
HTN, n (%)	9 (56.25)	9 (69.23)	13 (40.63)	26 (53.06)	5 (38.46)	62 (50.41)
DM, n (%)	9 (56.25)	8 (61.54)	14 (43.75)	19 (38.78)	4 (30.77)	54 (43.9)
CAD, n (%)	11 (68.75)	3 (23.08)	10 (31.25)	23 (46.94)	1 (7.69)	48 (39.02)
CKD, n (%)	4 (25)	1 (7.69)	8 (25)	7 (14.29)	1 (7.69)	21 (17.07)
**ECG feature**						
HR, bpm	75.50 ± 11.26	88.00 ± 12.45	74.88 ± 12.70	67.65 ± 9.86	101.08 ± 18.47	76.24 ± 15.95

PR interval, ms	181.62 ± 30.09	192.86 ± 21.57	177.33 ± 37.00	182.12 ± 45.31	170.60 ± 27.01	180.99 ± 38.28
QT interval, ms	421.38 ± 38.45	397.69 ± 39.58	427.09 ± 36.59	422.02 ± 40.80	373.38 ± 42.77	415.54 ± 42.31
QRS duration, ms	112.38 ± 20.66	122.62 ± 31.94	119.78 ± 25.13	111.80 ± 26.70	108.23 ± 30.50	114.72 ± 26.59
QTc interval, ms	469.56 ± 32.62	477.92 ± 24.92	473.81 ± 38.85	445.37 ± 39.42	479.08 ± 35.57	462.92 ± 39.03
P axis	53.44 ± 20.28	54.14 ± 15.81	58.05 ± 23.01	43.56 ± 32.23	39.00 ± 67.46	49.36 ± 30.28
R axis	41.62 ± 32.86	17.00 ± 48.85	22.50 ± 64.52	19.29 ± 59.53	17.69 ± 63.84	22.62 ± 57.26
T axis	97.87 ± 50.35	57.08 ± 69.09	77.00 ± 103.26	90.59 ± 82.26	39.23 ± 36.59	78.91 ± 81.05
**ECG LVSD feature**						
Prolonged QRS	8 (50)	5 (38.46)	16 (50)	14 (28.57)	3 (23.08)	46 (37.40)
LAD	2 (12.50)	4 (30.77)	8 (25)	13 (26.53)	4 (30.77)	31 (25.20)
RAD	1 (6.25)	0 (0)	4 (12.50)	7 (14.29)	2 (15.38)	14 (11.38)
QT prolongation	11 (68.75)	11 (84.62)	23 (71.88)	16 (32.65)	10 (76.92)	71 (57.72)

Abnormal QRS-T axis	0 (0)	1 (7.69)	5 (15.62)	5 (10.20)	0 (0)	11 (8.94)
delayed ID	9 (56.25)	5 (38.46)	12 (37.50)	23 (46.94)	3 (23.08)	52 (42.28)
Abnormal Q						
V2, V3, V4	4 (25.00)	0 (0)	1 (3.12)	10 (20.41)	0 (0)	15 (12.20)
V1, V2	5 (31.25)	0 (0)	2 (6.25)	12 (24.49)	0 (0)	19 (15.45)
V5, V6, I, aVL	1 (6.25)	0 (0)	2 (6.25)	3 (6.12)	0 (0)	6 (4.88)
II, aVF, III	1 (6.25)	0 (0)	4 (12.50)	7 (14.29)	1 (7.69)	13 (10.57)
T wave inversion						
I, V5, or V6	5 (31.25)	0 (0)	7 (21.88)	7 (14.29)	1 (7.69)	20 (16.26)
V2-4	5 (31.25)	0 (0)	2 (6.25)	4 (8.16)	2 (15.38)	13 (10.57)
II, aVF	0 (0)	0 (0)	4 (12.50)	1 (2.04)	0 (0)	5 (4.07)
ST elevation						
V2-4	3 (18.75)	4 (30.77)	3 (9.38)	8 (16.33)	1 (7.69)	19 (15.45)
V1-2	0 (0)	2 (15.38)	2 (6.25)	4 (8.16)	1 (7.69)	9 (7.32)
V5, V6, I, aVL	0 (0)	0 (0)	0 (0)	0 (0)	0 (0)	0 (0)
II, III, aVF	0 (0)	0 (0)	0 (0)	0 (0)	0 (0)	0 (0)

ST depression						
V2-4	0 (0)	0 (0)	3 (9.38)	0 (0)	1 (7.69)	4 (3.25)
V1-2	0 (0)	0 (0)	0 (0)	0 (0)	1 (7.69)	1 (0.81)
V5, V6, I, aVL	0 (0)	0 (0)	8 (25.00)	0 (0)	1 (7.69)	9 (7.32)
II, III, aVF	0 (0)	0 (0)	1 (3.12)	1 (2.04)	0 (0)	2 (1.63)
Tall T						
V2-4	1 (6.25)	3 (23.08)	2 (6.25)	1 (2.04)	1 (7.69)	8 (6.50)
V1-2	0 (0)	2 (15.38)	1 (3.12)	0 (0)	1 (7.69)	4 (3.25)
V5, V6, I, aVL	0 (0)	0 (0)	0 (0)	0 (0)	0 (0)	0 (0)
II, III, aVF	0 (0)	0 (0)	0 (0)	1 (2.04)	0 (0)	1 (0.81)
**ECG device interpretation**						
SR	16 (100)	8 (61.54)	22 (68.75)	34 (69.39)	5 (38.46)	85 (69.11)
LVH	2 (12.5)	2 (15.38)	12 (37.50)	6 (12.24)	5 (38.46)	27 (21.95)
AF	0 (0)	5 (38.46)	7 (21.88)	7 (14.29)	7 (53.85)	26 (21.14)
LBBB	1 (6.25)	2 (15.38)	1 (3.12)	4 (8.16)	1 (7.69)	9 (7.32)
RBBB	1 (6.25)	1 (7.69)	3 (9.38)	0 (0)	2 (15.38)	7 (5.69)
PM	0 (0)	0 (0)	2 (6.25)	4 (8.16)	0 (0)	6 (4.88)
**Echocardiographic findings**						
EF, %	36.75 ± 8.55	41.46 ± 13.22	35.97 ± 11.41	39.37 ± 11.05	38.19 ± 11.55	38.24 ± 11.12

LVIDs, mm	42.90 ± 10.20	45.23 ± 13.53	48.76 ±. 0.77	44.48 ± 9.90	43.36 ± 12.00	45.46 ± 10.88
LVIDd, mm	59.20 ± 8.64	58.31 ± 11.02	60.63 ± 8.64	57.80 ± 8.66	53.40 ± 9.29	58.33 ± 9.09
Mitral inflow E cm/s	63.25 ± 26.09	87.17 ± 17.72	86.52 ± 37.22	68.40 ± 21.12	67.25 ± 22.58	75.01 ± 27.40
Mitral inflow A cm/s	68.38 ± 22.60	83.67 ± 15.59	67.64 ± 22.84	72.83 ± 24.68	70.67 ± 30.66	71.86 ± 23.19
Deceleration time, ms	210.88 ± 53.98	201.82 ± 83.70	204.93 ± 83.34	217.89 ± 56.21	150.88 ± 58.94	205.34 ± 69.03
Septal E′ cm/s	4.54 ± 1.85	11.58 ± 20.46	4.23 ± 1.88	4.97 ± 1.60	5.65 ± 2.02	5.76 ± 7.99
Septal A′ cm/s	7.41 ± 2.17	6.83 ± 2.14	5.88 ±. .83	6.95 ± 2.10	6.33 ± 2.52	6.69 ± 2.32
E/E′ ratio	17.51 ± 12.83	18.34 ± 12.85	21.71 ± 9.69	15.52 ± 8.27	12.59 ± 3.39	17.38 ± 9.79
E/A ratio	1.27 ± 1.41	1.04 ± 0.21	1.40 ± 0.50	1.10 ± 0.77	0.87 ± 0.42	1.18 ± 0.90
PASP, mmHg	31.50 ± 14.65	29.15 ± 8.15	30.96 ± 13.92	26.18 ± 8.86	23.80 ± 4.71	28.17 ± 10.89
**LVSD score and reference standard**						
AiTiALVSD score	42.98 ± 23.20	33.24 ± 25.71	55.87 ± 28.50	40.01 ± 22.43	37.75 ± 26.41	43.57 ± 25.77

LVSD (True positive)	13 (81.25)	8 (61.54)	24 (75)	33 (67.35)	10 (76.92)	88 (71.54)
non-LVSD (False positive	3 (18.75)	5 (38.46)	8 (25)	16 (32.65)	3 (23.08)	35 (28.46)

Values are expressed as n(%), mean ± standard deviation; HTN, hypertension; DM, diabetes mellitus; CAD, coronary artery disease; CKD, chronic kidney disease; SR, sinus rhythm; AF, atrial fibrillation; PM, pacemaker; LVH, left ventricular hypertrophy; BBB, right and left bundle branch block

Supplementary Table S6. Diagnostic performance of AiTiALVSD; 12 types of lead reversal ECGs

	AUROC (95% CI)	AUPRC (95% CI)	Sensitivity (95% CI)	Specificity (95% CI)	PPV (95% CI)	NPV (95% CI)
1. [Nonreversal] or [LL/RL reversal]	0.971 (0.954-0.989)	0.883 (0.838-0.923)	0.898 (0.838-0.958)	0.940 (0.921-0.959)	0.715 (0.636-0.795)	0.982 (0.971-0.993)
2. [LL/LA reversal] or [Counter clockwise reversal w/o RA]	0.965 (0.950-0.980)	0.834 (0.774-0.885)	0.908 (0.851-0.965)	0.892 (0.867-0.917)	0.586 (0.507-0.664)	0.983 (0.972-0.994)
3.[Clockwise w/o RA] or [RL/LA reversal]	0.966 (0.951-0.980)	0.824 (0.760-0.883)	0.867 (0.800-0.935)	0.925 (0.903-0.946)	0.659 (0.577-0.741)	0.976 (0.964-0.989)
4. [RA/LA reversal] or [RA/LA and RL/LL reversal]	0.940 (0.912-0.967)	0.811 (0.751-0.866)	0.827 (0.752-0.901)	0.938 (0.919-0.958)	0.692 (0.609-0.776)	0.970 (0.956-0.984)
5. [Clockwise reversal w/o RL] or [Counter clockwise]	0.921 (0.891-0.952)	0.724 (0.647-0.795)	0.847 (0.776-0.918)	0.870 (0.842-0.897)	0.522 (0.444-0.600)	0.971 (0.957-0.986)
6. [RA>LA, LA>RL, LL>RA, RL>LL] or	0.949 (0.929-0.969)	0.788 (0.726-0.851)	0.806 (0.728-0.884)	0.931 (0.911-0.952)	0.664 (0.579-0.749)	0.966 (0.951-0.981)

[Clockwise reversal w/o LL]						
7. [RA/LL reversal] or [Counter clockwise reversal w/o LA]	0.893 (0.848-0.937)	0.741 (0.667-0.806)	0.888 (0.825-0.950)	0.708 (0.672-0.745)	0.339 (0.281-0.396)	0.974 (0.959-0.989)
8. [Clockwise reversal w/o RL] or [RA>LL, LA>RA, LL>RL, RL>LA]	0.910 (0.874-0.945)	0.735 (0.667-0.798)	0.939 (0.891-0.986)	0.611 (0.571-0.650)	0.288 (0.239-0.338)	0.983 (0.970-0.997)
9. [RA>LL, LA>RL, LL>LA, RL>RA] or [RA/LL and LA/RL reversal]	0.903 (0.864-0.943)	0.730 (0.656-0.801)	0.898 (0.838-0.958)	0.698 (0.661-0.735)	0.333 (0.276-0.390)	0.976 (0.961-0.991)
10. [RA/RL reversal] or [Clockwise reversal w/o LA]	0.942 (0.916-0.968)	0.816 (0.758-0.868)	0.918 (0.864-0.973)	0.822 (0.791-0.853)	0.464 (0.394-0.534)	0.984 (0.972-0.995)
11. [RA/RL and LA/LL reversal] or [RA>RA, LA>LL, LL>RA, RL>LA reversal]	0.923 (0.892-0.954)	0.744 (0.664-0.812)	0.929 (0.878-0.980)	0.695 (0.657-0.732)	0.338 (0.282-0.395)	0.983 (0.971-0.995)

12.[Clockwise reversal] or [Clockwise reversal w/o LL]	0.932 (0.903-0.962)	0.797 (0.734-0.854)	0.857 (0.788-0.926)	0.883 (0.857-0.909)	0.553 (0.474-0.632)	0.974 (0.960-0.987)

AUROC, area under the receiver operating characteristic curve; AUPRC, area under the precision-recall curve; LA, left arm; RA, right arm; LL, left leg; RL, right leg; w/o, without

Supplementary Table S7. Diagnostic performance of AiTiALVSD; 6 types of ECG noise

	**AUROC (95% CI)**	**AUPRC (95% CI)**	**Sensitivity (95% CI)**	**Specificity (95% CI)**	**PPV (95% CI)**	**NPV (95% CI)**
**Power-line Noise**	0.962 (0.942-0.977)	0.838 (0.767-0.897)	0.812 (0.734-0.885)	0.944 (0.923-0.963)	0.713 (0.618-0.797)	0.967 (0.951-0.981)
**EMG Noise**	0.950 (0.926-0.970)	0.813 (0.729-0.878)	0.921 (0.857-0.968)	0.847 (0.815-0.873)	0.508 (0.434-0.570)	0.984 (0.971-0.994)
**Baseline Wander**	0.974 (0.958-0.985)	0.880 (0.826-0.926)	0.861 (0.793-0.921)	0.944 (0.924-0.963)	0.725 (0.642-0.802)	0.975 (0.963-0.987)
**Baseline Shift**	0.975 (0.959-0.987)	0.891 (0.840-0.934)	0.901 (0.833-0.956)	0.940 (0.921-0.958)	0.722 (0.642-0.793)	0.982 (0.970-0.993)
**Partial White Noise**	0.971 (0.955-0.985)	0.880 (0.825-0.926)	0.901 (0.840-0.958)	0.932 (0.912-0.951)	0.695 (0.610-0.772)	0.982 (0.971-0.993)
**Time Mask**	0.969 (0.947-0.984)	0.888 (0.834-0.931)	0.901 (0.835-0.953)	0.935 (0.916-0.954)	0.705 (0.623-0.777)	0.982 (0.970-0.991)

AUROC, area under the receiver operating characteristic curve; AUPRC, area under the precision-recall curve; PPV, positive predictive value; NPV, negative predictive value; CI, confidence interval.

Supplementary Table S8. TCAV scores with 95% confidence intervals by feature block and concept

	LAD	RAD	LBBB	RBBB	AF	Abnormal Q wave	Abnormal T wave	Prolonged QT interval	Conduction disorder	Random control
**Feature block 1**	0.816 (0.784 – 0.848)	0.797 (0.755 – 0.839)	0.984 (0.978 – 0.990)	0.937 (0.917 – 0.957)	0.974 (0.966 – 0.982)	0.926 (0.906 – 0.946)	0.964 (0.957 – 0.971)	0.992 (0.989 – 0.995)	0.947 (0.938 – 0.956)	0.074 (0.065 – 0.083)
**Feature block 2**	0.732 (0.696 – 0.768)	0.580 (0.535 – 0.625)	0.844 (0.803 – 0.885)	0.767 (0.719 – 0.815)	0.939 (0.919 – 0.959)	0.660 (0.619 – 0.701)	0.664 (0.640 – 0.688)	0.573 (0.526 – 0.620)	0.897 (0.855 – 0.939)	0.260 (0.243 – 0.277)
**Feature block 3**	0.676 (0.597 – 0.755)	0.413 (0.327 – 0.499)	0.946 (0.930 – 0.962)	0.727 (0.637 – 0.817)	0.800 (0.707 – 0.893)	0.663 (0.535 – 0.791)	0.470 (0.349 – 0.591)	0.462 (0.367 – 0.557)	0.747 (0.620 – 0.874)	0.344 (0.311 – 0.377)
**Feature block 4**	0.703 (0.600 – 0.806)	0.969 (0.939 – 0.999)	0.900 (0.836 – 0.964)	0.993 (0.990 – 0.996)	0.973 (0.971 – 0.975)	0.883 (0.797 – 0.969)	0.960 (0.950 – 0.970)	0.512 (0.423 – 0.601)	0.968 (0.962 – 0.974)	0.126 (0.103 – 0.149)
**Average TCAV score for total blocks**	0.731	0.689	0.918	0.856	0.921	0.783	0.764	0.634	0.889	0.201

LVSD, left ventricular systolic dysfunction; ECG, electrocardiogram; LAD, left axis deviation; RAD, right axis deviation; LBBB, left bundle branch block; RBBB, right bundle branch block; AF, atrial fibrillation.

Supplementary Text

Supplementary Text S1. AiTiALVSD algorithm development

We developed a model based on a residual neural network (ResNet) utilizing the PyTorch library and Python for programming. ResNet, typically used for image processing, works by utilizing convolutions to identify intricate patterns within datasets. ECG classifications possess complex characteristics for classification tasks in image and time series domains. Such tasks require extracting rhythm and morphological features from ECG. Figure S1.1 shows a structure of our model. We employed architectures with a stem block, 12 residual blocks, and a single fully connected network to detect patterns in these features. Each block extracts the features of the ECG and passes them to the next block. We defined a feature block as a group of three residual blocks where AiTiALVSD contains four feature blocks. The residual block consists of a set of layers: a one-dimensional convolutional neural network, batch normalization, rectified linear unit (ReLU) activations, another one-dimensional convolutional neural network, additional batch normalization, a subsequent ReLU activation, a dropout layer and skip connection. The stem block contains a single layer with a skip connection and max pooling.

Our ECG data were collected from three hospitals in the Republic of Korea, excluding the hospital utilized in this study. All digitalized 12-lead ECGs were originally measured at either 500 Hz or 250 Hz for 10 seconds. To standardize the input format and enhance model efficiency, all signals were resampled to 250 Hz—downsampling the 500 Hz data accordingly. This allowed us to reduce the computational burden and model size while preserving relevant diagnostic information. Each ECG was then represented as a 12×2500 matrix for input into the model. Subsequently, we applied a bandpass filter (0.5–120 Hz) to eliminate baseline drift and high-frequency noise.Additionally, we used a bandpass filter with a frequency range of 0.5–120 Hz to remove baseline drift and high-frequency noise.

An example illustrating each stage of preprocessing—including raw signal, resampled signal, and filtered output—is provided in Supplementary Figure S1.2. Additionally, we employed data augmentation to enhance the model's generalization. Our dataset was split into three parts, with 80% of the data used for training, 15% for validation during training, and 5% for testing the final model. ECGs from a single patient were not split. The positive label was defined as an ejection fraction (EF) of 40% or less as observed on cardiac ultrasound by the diagnostic and treatment guidelines for heart failure presented by the European Society of Cardiology. Conversely, the zero labels indicated an EF of greater than 40%.

We initialized the model parameters using random initialization following a normal distribution. The final model has 9,777,284 trainable parameters. For model training, we used the Adam optimizer in combination with a cosine warm-up learning rate scheduler and the focal loss function. To optimize the model architecture and hyperparameters, we employed a population-based training scheduler. The search space included the number of feature blocks [2, 3, 4], number of layers per block, [2, 3, 4], convolution kernel sizes [7, 9, 11], dropout rates [0.2, 0.5], learning rates [0.0001, 0.00001, 0.000001], and weight decay values [0.0001, 0.000001]. We trained the model for 150 epochs across these configurations and selected the best-performing model based on the highest combined AUROC and AUPRC on the validation set. To prevent overfitting and optimize training efficiency, we incorporated an early stopping strategy based on the Population-Based Training (PBT) algorithm, which dynamically stops underperforming trials if no performance improvement is observed. As shown in Figure S1.3, in our experiments, the validation performance began to degrade beyond epoch 37 due to overfitting. Therefore, we selected the model at epoch 37 to preserve generalization performance.

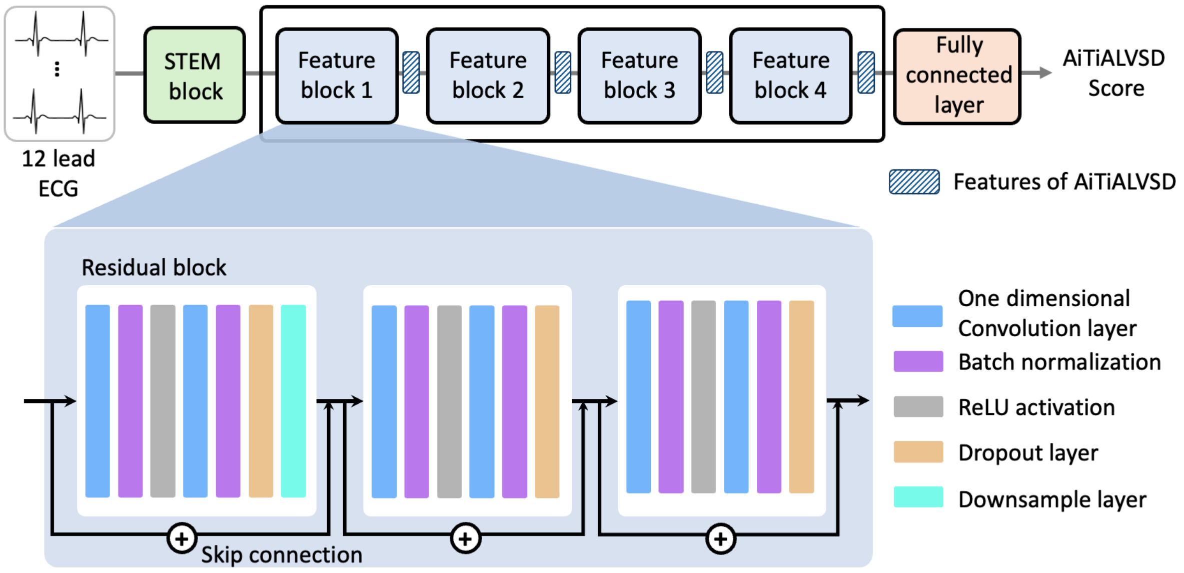

Figure S1.1 Structure of AiTiALVSD. It shows structure of AiTiALVSD and process to compute an AiTiALVSD score. The process begins with a 12-lead ECG input which goes through a STEM block, followed by four sequential feature blocks, and finally culminates in a fully connected layer that outputs the AITIALVSD score. Each feature block contains three residual blocks with skip connections. Inside the residual blocks, there are layers represented by colored bars, each color corresponding to a different type of operation: one-dimensional convolution layers, batch normalization, ReLU activation functions, dropout layers, and downsample layer.

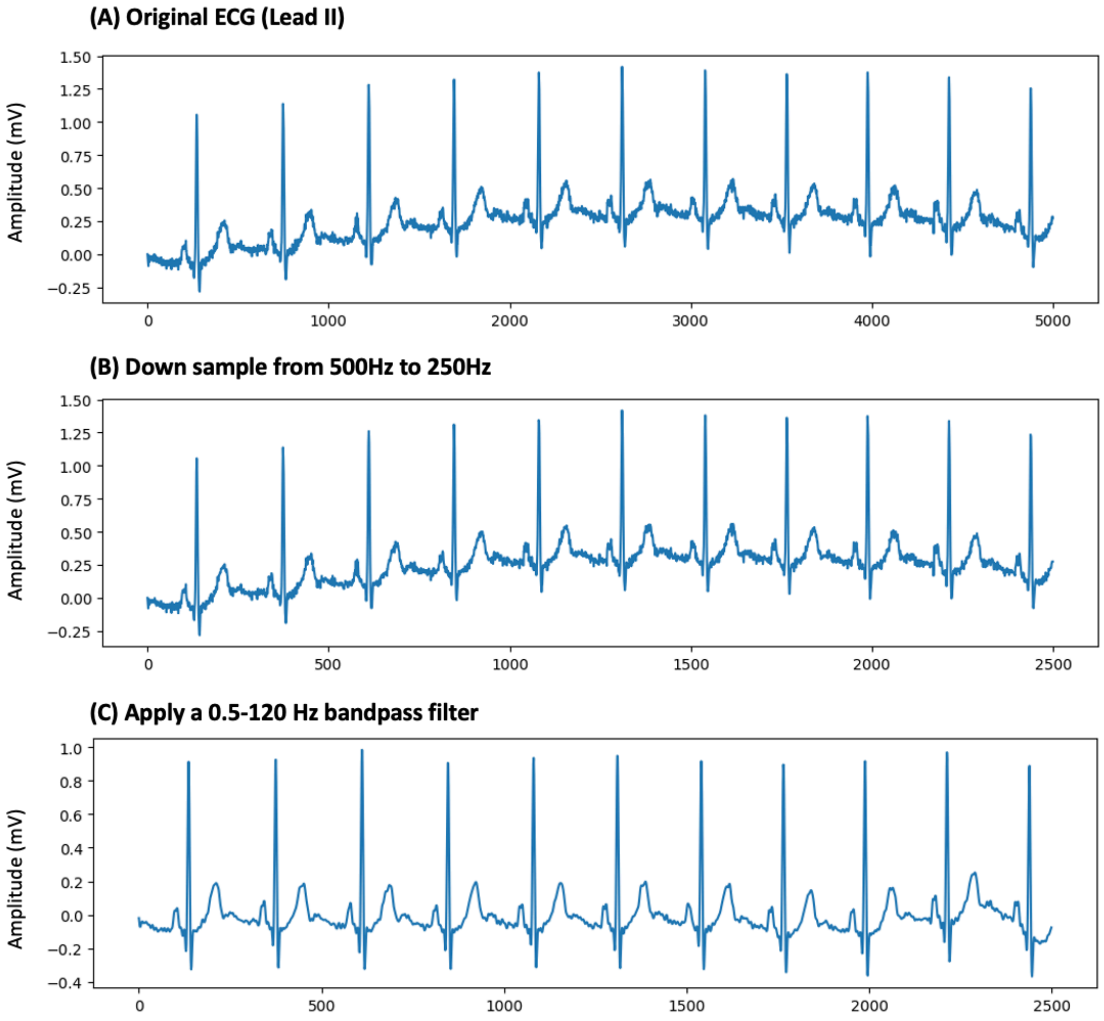

Figure S1.2. Example of ECG preprocessing steps. This figure illustrates the sequential preprocessing steps applied to a 12-lead ECG signal: (A) the original raw signal sampled at 500 Hz, (B) the resampled signal from 500 Hz to 250 Hz, and (C) the final signal after applying a bandpass filter (0.5–120 Hz). These steps were performed to standardize input data and reduce noise prior to model training.

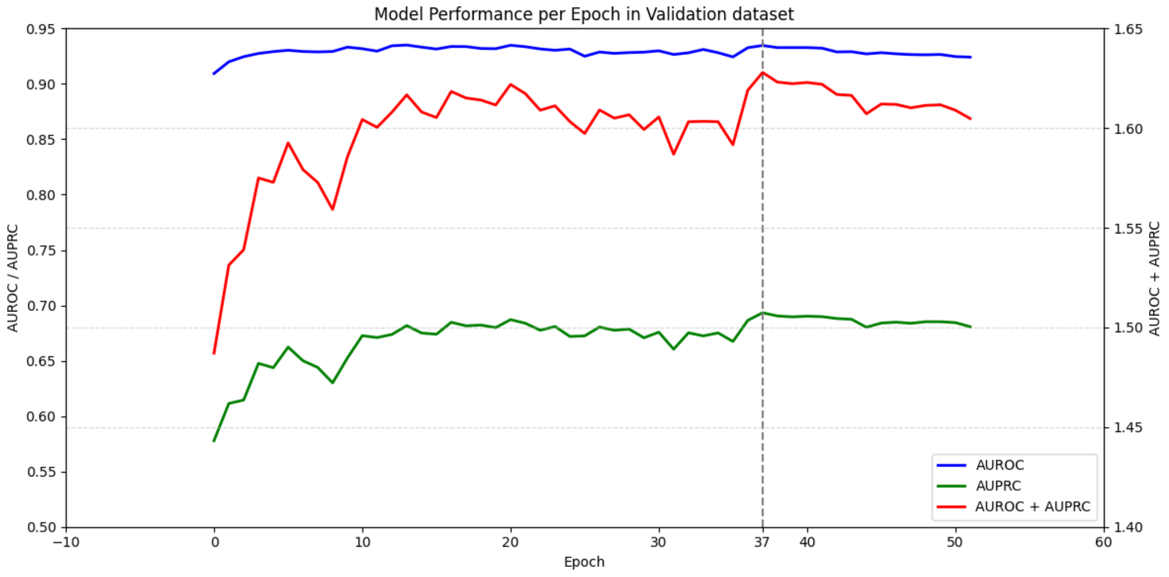

Figure S1.3 Validation performance across training epochs. It shows AUROC (blue), AUPRC (green), and their combined score (AUROC + AUPRC, red) evaluated on the validation dataset. The combined score reached its peak at epoch 37 (indicated by the vertical dashed line), which was selected as the optimal model checkpoint to preserve generalization performance and prevent overfitting.

Supplementary Text S2. Model Card of AiTiALVSD for model development

Table S2.1 shows demographic distribution of dataset for AiTiALVSD development. This dataset consists of 353,845 records, divided into 291,564 training entries (79.9% of the total), 55,320 validation entries (15.1%), and 17,961 testing entries (5.0%).

Table S2.1 Data distribution of AiTiALVSD development

	**Train dataset**	**Validation dataset**	**Test dataset**	**Total**
**# of ECG record**	291564	55320	17961	364845
**Hospitals**				
**Hospital A**	193702	37074	11883	242659
**Hospital B**	60622	11353	3737	75712
**Hospital C**	22053	4019	1378	27450
**Hospital D**	15187	2874	963	19024
**Gender**				
**Female**	124277	23333	7688	155298
**Male**	162139	30945	9954	203038
**Unknown**	5148	1042	319	6509
**Age group**				
	69	14	4	87
**~20**	1683	294	97	2074
**20~30**	7828	1393	555	9776
**30~40**	14944	2854	1002	18800
**40~50**	32415	6167	1937	40519
**50~60**	57633	10552	3934	72119

**60~70**	66647	12491	3926	83064
**70~80**	69352	13345	4047	86744
**80~90**	31864	6351	1968	40183
**90~100**	3045	636	132	3813
**100~**	44	4	0	48
	0	1	0	1
	1	0	0	1

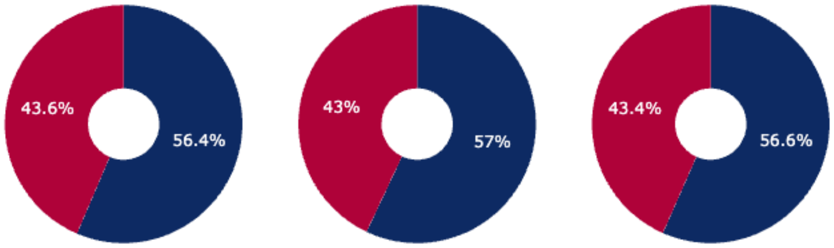

Figure S2.1 Proportion of gender in datasets.

Gender distribution within the dataset is detailed as follows: females account for 124,277 records in the training set (43.6%), 23,333 in validation (43.0%), and 7,688 in testing (43.4%). Males are represented by 162,139 records in the training set (56.4%), 30,945 in validation (57.0%), and 9,954 in testing (56.6%). Unknown gender records comprise 5,148 in training, 1,042 in validation, and 319 in testing.

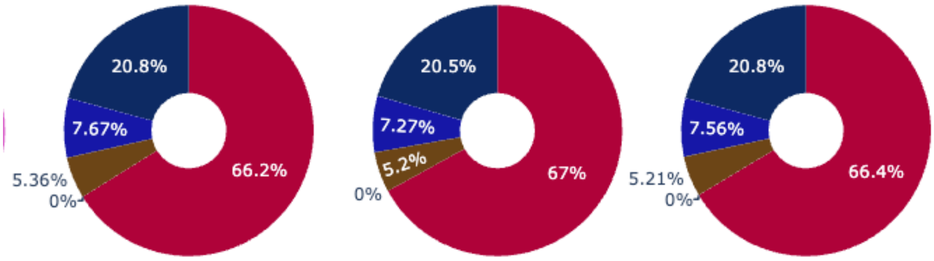

Figure S2.2 Proportion of hospital in each dataset.

Hospital affiliation data is categorized across four hospitals: Hospital A, B, C, and D. Hospital A is the most represented with 193,702 records in the training set (66.2%), 37,074 in validation (67.0%), and 11,883 in testing (66.4%). Hospital B contributes 60,622 records to training (20.8%), 11,353 to validation (20.5%), and 3,737 to testing (20.8%). Hospital C and D provide smaller but significant contributions, ensuring a diverse range of medical practices and patient demographics are represented, enhancing the dataset's applicability to various research contexts.

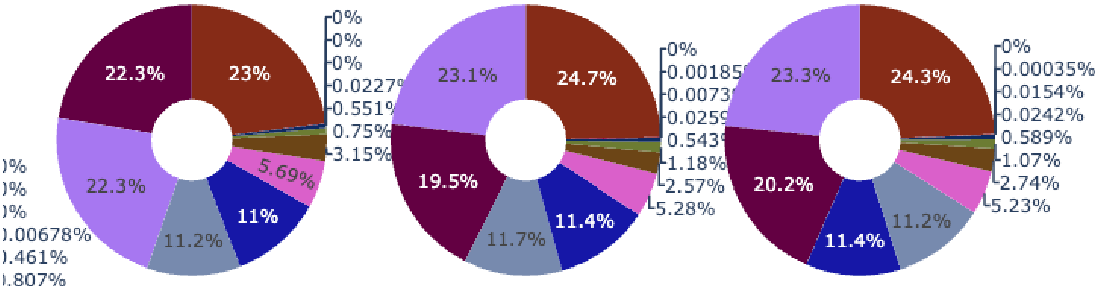

Figure S2.2 Proportion of age group in each dataset.

The age group distribution is notably skewed towards older individuals, particularly beneficial for research on age-related conditions. The most represented age groups are 50-80 years, with the 70-80 age group alone accounting for 4,047 records in training (1.4%), 13,345 in validation (24.1%), and 69,352 in testing (386.3%).

In summary, the dataset's composition—characterized by a balanced gender distribution, diverse hospital affiliations, and a focus on broad age groups—offers a solid foundation for a wide range of medical research topics. The proportional representation of different demographics and affiliations within the dataset underscores its potential for generating nuanced and broadly applicable health insights.

The dataset's structure and diversity are conducive to a wide array of medical research topics, from gender and age-related studies to analyses based on hospital-specific data. Its

comprehensive nature allows for the development of nuanced and broadly applicable health insights.

Supplementary Text S3. Interpretation methods for model transparency

A. Concept-based Method: Test concept activation vector (TCAV)

TCAV is a technique for interpreting the decisions made by a deep neural network. It is designed to provide insights into the internal workings of a model in a way that is understandable to humans.

The main idea behind TCAV is to represent high-level concepts as vectors in the activation space of a neural network layer. These vectors, known as concept activation vectors (CAVs), can be used to measure the extent to which a given concept is utilized by the model for prediction. By calculating a TCAV score for a concept, we can quantify the overall importance of that concept for the decisions made by the model. Figure S3.1 shows a process of TCAV. Below is the detailed process of TCAV we applied.

1. **Concept Identification:** The first step in TCAV involves identifying the concepts of interest. These concepts can be anything that you believe might have an influence on the decisions made by the model. For example, in an image classification task, concepts could be colors, textures, shapes, or even specific objects. We utilized the Physionet open dataset to define concepts and construct a concept dataset. The Physionet challenge dataset is an integrated dataset of ECGs collected from six different hospitals. The Physionet dataset comprises a total of N ECGs, each labeled with one of 26 arrhythmias, making it a multilabel ECG dataset. Each ECG possesses one or more arrhythmia labels. Figure S3.1 displays the label distribution of the Physionet dataset.

   We defined nine target concepts using these labels. Our objective was to ascertain whether AiTiALVSD positively utilizes these target concepts in determining LVSD by using the TCAV score. The nine concepts were as follows: atrial fibrillation and atrial flutter (AF), left bundle branch block (LBBB), right bundle branch block

(RBBB), left axis deviation (LAD), right axis deviation (RAD), prolonged QT, nonspecific intraventricular conduction disorder (conduction disorder), and combined abnormal q wave and abnormal t wave (abnormal Q, T wave). The AF and abnormal Q wave concepts were defined by combining two labels. The AF concept included both atrial fibrillation and atrial flutter. The abnormal Q wave concept included both abnormal T wave and T wave inversion labels.

In constructing the concept datasets, we ensured that there were no overlapping ECGs among them by extracting distinct ECGs. From the remaining labels not used for the concepts, we randomly selected approximately 200 samples to create 10 random concept datasets. When constructing a concept dataset, we ensured that the number of data extracted from each data source was balanced. This was to prevent any specific concept dataset from having a bias toward a particular data source. If a data source was lacking labels, we supplemented it with additional data from another source. Table S1 shows the distribution of data sources in each concept dataset. As a result, we established nine target concept datasets and 10 random concept datasets.

2. **Feature Extraction:** Once the concepts are identified, the next step is to extract hidden features of each block. Figure S3.1 (2) shows an example of hidden feature extraction from the first block of AiTiALVSD. Following a feature representation for AiTiALVSD to predict LVSD, the features of all samples from the concept datasets are extracted.
3. **Concept Activation Vector (CAV):** A Concept Activation Vector is a direction in the activation space of a neural network layer that represents a concept. To create a CAV, a linear classifier is trained (such as a logistic regression or SVM) to distinguish between the above two sets of targets that can random concept features. The vector orthogonal to the decision boundary of this classifier is defined as the CAV for the

target concept.

4. **Extraction feature from positive LVSD samples:** features of 300 positive LVSD samples are extracted using the same process as step (2).

5. **Testing with CAVs (TCAV Score Calculation):** After the CAV and positive LVSD features are created, they can be used to interpret the decisions made by the model. For given LVSD features, it moves slightly onto the CAV. We check whether the moved LVSD feature of the AiTiALVSD score increases when predicted again. By repeating this process for all LVSD samples and averaging the results, a TCAV score for the target concept is calculated as follows: This score quantifies the overall importance of the concept for the decisions made by the model.

$$\text{TCAV score of target concept} = \frac{\#\ of\ increased\ score\ sample}{total\ \#\ of\ samples}$$

6. **Interpretation:** If a concept has a high TCAV score, it means that the model places much positive importance on that concept when making decisions. If a concept has a low TCAV score, it means that the concept is not important for the model's decisions.

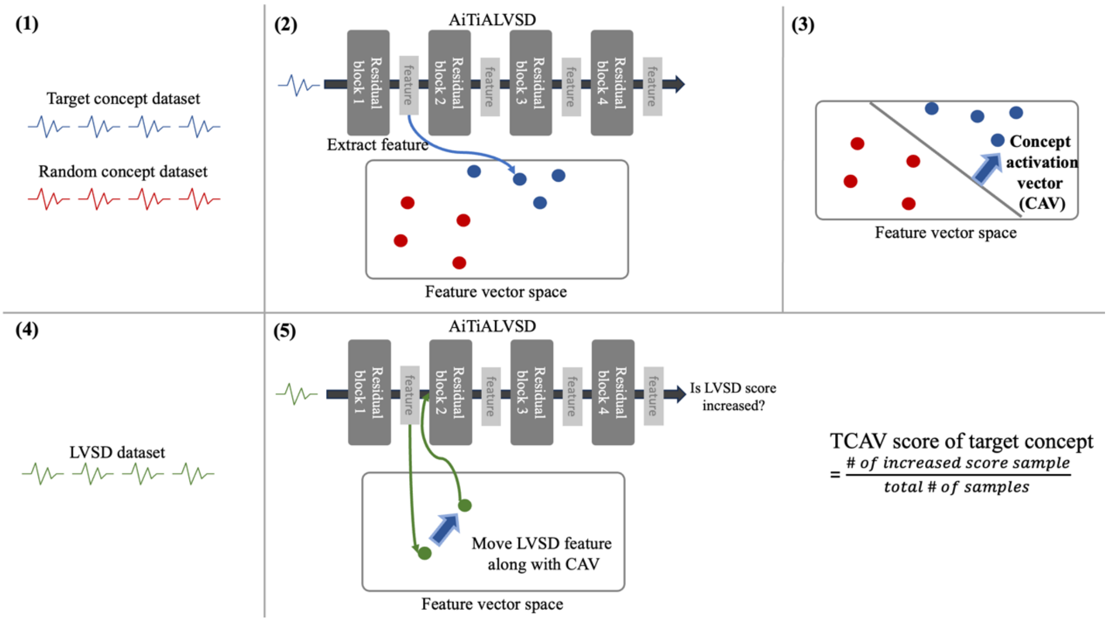

Figure S3.1 Process of TCAV

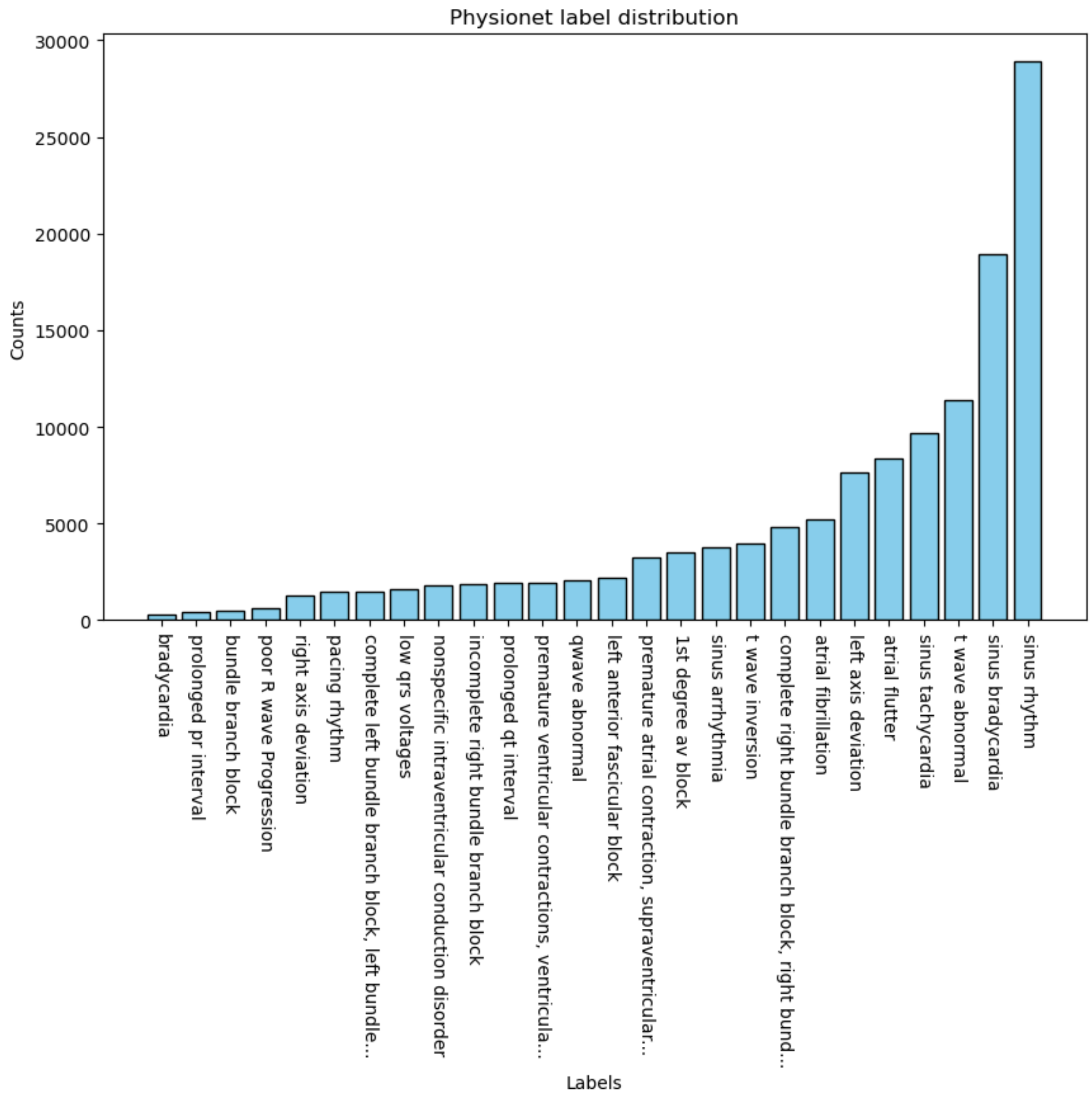

Figure S3.2 Histogram of label count in the Physionet dataset

Table S3.1 Distribution of data sources for each concept dataset

	**Ningbo**	**Georgia**	**Chapman Shaoxing**	**PTBXL**	**CPSC2018**	**CPSC2018 extra**
**right axis deviation**	79	40	40	40	-	1
**complete left bundle branch**	33	33	33	63	33	5

**block, left bundle...**						
**nonspecific intraventricular conduction disorder**	40	40	40	76	-	4
**prolonged QT interval**	40	76	40	40	-	4
**Q wave abnormal**	79	40	40	40	-	1.0
**complete right bundle branch block, right bund...**	33	33	33	33	35	33
**left axis deviation**	50	50	50	50	-	-
**atrial fibrillation+atrial flutter**	35	33	33	33	33	33
**T wave abnormal + T wave inversion**	54	40	40	40	-	26
**random_concept_0**	33	33	33	33	33	33

**random_concept_1**	33	33	33	33	33	33
**random_concept_2**	33	33	33	33	33	33
**random_concept_3**	33	33	33	33	33	33
**random_concept_4**	33	33	33	33	33	33
**random_concept_5**	33	33	33	33	33	33
**random_concept_6**	33	33	33	33	33	33
**random_concept_7**	33	33	33	33	33	33
**random_concept_8**	33	33	33	33	33	33
**random_concept_9**	33	33	33	33	33	33

Ningbo, Ningbo Database; Georgia, The Georgia 12-lead ECG Challenge (G12EC) Database; PTB, PTB and PTB-XL Database

Supplementary Text S4. Sample size calculation

In the prior studies that developed the software, the respective AUROCs were ranging from 0.877 to 0.9790.925 and 0.963; however, considering the possibility of overestimation in this study. We assumed an expected AUROC of 0.85 for the strict clinical trial, accounting for the

potential for limited performance. Using the AUROC values of BNP or NT-proBNP, the conventional representative indicators for screening LVSD, as the basis for determining the minimum clinical performance standard, the reported performance levels range widely from 0.56 to 0.90. Consequently, we set the validity evaluation criteria for this clinical trial at AUROC 0.75 and determined a superiority margin of 0.1 to calculate the sample size. Furthermore, since the prevalence of LVSD varies among communities and hospitals, we set the prevalence at 15%. Sample size calculation was performed using PASS 15 (NCSS, LLC. Kaysville, USA) software. The performance estimate of AiTiALVSD was set at AUROC 0.85, the clinical validity evaluation criterion at AUROC 0.75, and the significance level (α) and statistical power ($1-\beta$) at 0.05 and 95%, respectively. Considering a dropout rate of 5%, the total sample size was 688 participants (103 LVSD and 585 Non-LVSD).

B. Cluster Analysis

We performed clustering analysis to analyze the characteristics of LVSD that AiTiALVSD recognizes. By comparing the ECG features of each cluster group and visually verifying them, we aimed to distinguish specific ECG differences. The clustering algorithm was executed using the sklearn python package version 1.1.1. The details of the clustering analysis are as follows:

1. Selection of ECG samples and AiTiALVSD features: To understand the patterns of ECGs positively predicted by AiTiALVSD, we only used positively predicted ECG samples. As a result, the cluster algorithm was trained on a total of 60,125 positively predicted ECG samples in the development dataset, and the clustering results were visualized using positive predicted samples from the clinical trial. We utilized features extracted from the first block of AiTiALVSD as input for clustering. Features from the first block consisted of 64 channels, each with a size of 313. To address the curse of dimensionality and computational resource issues, we averaged each channelwise aggregation, converting them into 64-sized features. We trained the cluster algorithm using these transformed 64-sized features.

2. Clustering algorithm and determining the number of clusters: For the cluster analysis, we employed the K-means algorithm. To determine the optimal number of clusters, we used the elbow method. We set the number of clusters to five, which is most appropriate to capture the inherent patterns within the data.

3. Result visualization: To visualize the results of clustering, we employed the t-distributed stochastic neighbor embedding (T-SNE) method for dimensionality reduction. Using T-SNE, we reduced the 64-sized features into 2D data and visualized the results through a scatter plot. Supplementary Figures S4.1 and S4.2 shows the clustering visualization results for both the training dataset and the clinical dataset. We observed

distinct clusters in the clinical trial dataset, which indicates that the AiTiALVSD features internal phenotypes based on ECG characteristics.

4. Selection of representative ECGs: We assumed that each cluster defines a phenotype and compared the baseline characteristics of each cluster using a t test. Furthermore, to select a representative ECG, we chose the ECG closest to the tcentroid of each cluster from the clinical trial dataset. Figures S4.1 and S4.2 display the phenotype ECG selected from each cluster in the training and clinical trial datasets.

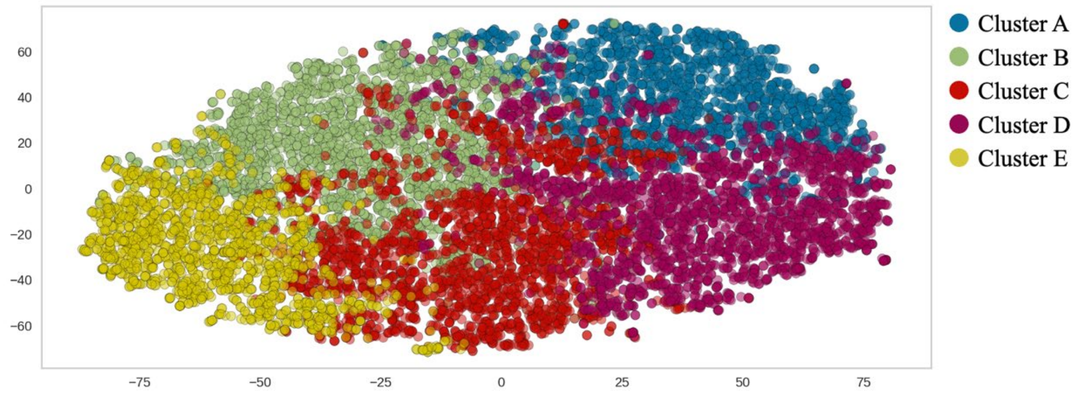

Figure S4.1 Clustering visualization using T-SNE in the development dataset

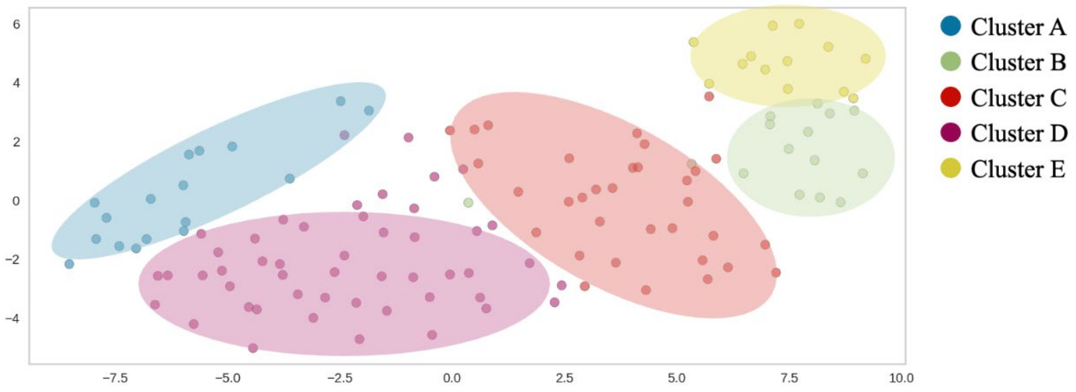

Figure S4.2 Clustering visualization using T-SNE in the clinical trial dataset
